# A pancreatic cancer organoid-in-matrix platform shows distinct sensitivities to T cell killing

**DOI:** 10.1038/s41598-024-60107-5

**Published:** 2024-04-23

**Authors:** Anton Lahusen, Jierui Cai, Reinhold Schirmbeck, Anton Wellstein, Alexander Kleger, Thomas Seufferlein, Tim Eiseler, Yuan-Na Lin

**Affiliations:** 1 Department of Internal Medicine I, Gastroenterology, Endocrinology, Nephrology, Nutrition and Metabolism, Ulm University Hospital, Albert Einstein Allee 23, 89081 Ulm, Germany; 2 Lombardi Comprehensive Cancer Center, Georgetown University, 3970 Reservoir Road NW, Washington, DC 20007 USA; 3 Institute of Molecular Oncology and Stem Cell Biology (IMOS), Ulm University Hospital, 89081 Ulm, Germany; 4 Division of Interdisciplinary Pancreatology, Department of Internal Medicine I, Ulm University Hospital, 89081 Ulm, Germany; 5 Organoid Core Facility, Ulm University Hospital, 89081 Ulm, Germany

**Keywords:** Cancer microenvironment, Cancer models, Tumour heterogeneity, Tumour immunology

## Abstract

Poor treatment responses of pancreatic ductal adenocarcinoma (PDAC) are in large part due to tumor heterogeneity and an immunosuppressive desmoplastic tumor stroma that impacts interactions with cells in the tumor microenvironment (TME). Thus, there is a pressing need for models to probe the contributions of cellular and noncellular crosstalk. Organoids are promising model systems with the potential to generate a plethora of data including phenotypic, transcriptomic and genomic characterization but still require improvements in culture conditions mimicking the TME. Here, we describe an INTERaction with Organoid-in-MatriX ("InterOMaX") model system, that presents a 3D co-culture-based platform for investigating matrix-dependent cellular crosstalk. We describe its potential to uncover new molecular mechanisms of T cell responses to murine KPC (*LSL-*Kras^*G12D*/+27^*/Trp53*^*tm1Tyj/J*^/p48^*Cre/*+^) PDAC cells as well as PDAC patient-derived organoids (PDOs). For this, a customizable matrix and homogenously sized organoid-in-matrix positioning of cancer cells were designed based on a standardized agarose microwell chip array system and established for co-culture with T cells and inclusion of stromal cells. We describe the detection and orthogonal analysis of murine and human PDAC cell populations with distinct sensitivity to T cell killing that is corroborated in vivo*.* By enabling both identification and validation of gene candidates for T cell resistance, this platform sets the stage for better mechanistic understanding of cancer cell-intrinsic resistance phenotypes in PDAC.

## Introduction

As one of the most aggressive malignancies, pancreatic ductal adenocarcinoma (PDAC) still poses a considerable challenge with limited therapeutic options^1,2^. This is largely due to its intratumor heterogeneity, a pronounced desmoplastic stroma contributing to the immunosuppressive tumor microenvironment (TME), as well as cancer cell-intrinsic drug and immune resistance mechanisms^3–6^. This resistance includes T cell modulatory therapies that have been highly successful in some cancers but are still of limited efficacy in PDAC^7–10^. To leverage chemo- and immunotherapeutic strategies for optimized personalized tumor-targeting therapies in PDAC, there is an urgent need to address cell–cell and cell–matrix crosstalk to understand cellular behavior in the context of their interplay and uncover underlying druggable vulnerabilities on a molecular level. PDAC cell-intrinsic factors shaping the immune TME were found to play a crucial role in antitumor immunity and lack of immunotherapy response^4–6^. Thus, there is an urgent need for appropriate model systems to pinpoint immune resistance mechanisms in PDAC cancer cell subpopulations. Patient-derived organoids (PDOs) have recently emerged in precision oncology due to their ability to recapitulate important characteristics of the original tumor specimen, including patient-specific responses to treatment^11,12^. However, major limitations of PDOs are their inability to fully recapitulate the microenvironment and tissue architecture, including the dynamic behavior inherent to cell function—major factors considerably impacting phenotypic screening results and clinical treatment response in patients^11–14^. Furthermore, most ex vivo immune cell-based co-culture assays disregard the role of the systemic immunity, thus ruling out the possibility of probing the potential impact of immunotherapeutic interventions with high fidelity^15^.

Previously, we reported on a 3D cancer cell spheroids/T cell co-culture model for monitoring T cell-mediated cancer cell killing using scaffold-free agarose microwell arrays^16^. This model system proved to be highly reliable in distinguishing between a T cell-sensitive and -resistant phenotype of clonal KPC (= LSL-Kras^G12D/+^/LSL-Trp53^R172H/+^/p48-Cre)^17^ PDAC cells directly co-cultured with PDAC tumor-educated T cells harvested from immunocompetent mice. By utilizing this model system, we unveiled underlying cancer PDAC cell-intrinsic C-X-C motif chemokine 12 (CXCL12) expression contributing to T cell-mediated cytotoxicity in a CXCL12/CXC chemokine receptor 4 (CXCR4)-dependent manner^3,16^. In this microarray setup, uniformly-sized PDAC spheroids are generated enabling high-throughput analysis of 3D co-culture studies under controlled and well-defined experimental conditions. However, a drawback of the system was the lack of monitoring T cell—cancer cell interactions through a matrix that mimics the microenvironment and can affect the crosstalk significantly^18^. Collagen type I is the most abundant component of the tumor extracellular matrix (ECM), and a strong correlation between its biophysical makeup, matrix stiffness and T cell activity and infiltration has been demonstrated^19,20^.

In the present study, we (i) enable the monitoring of interactions between cancer cells and T cells through a collagen matrix, (ii) employ murine PDAC spheroid lines as well as (iii) apply this to primary PDAC patient-derived organoid (PDO)-based cultures. Based on our previous co-culture platform, we constructed this more complex yet highly versatile 3D co-culture model system providing multiple complementary readouts to dissect the T cell response phenotype. We show that this model allows to probe cellular crosstalk in a scalable ex vivo system, which is well-defined and highly reproducible. We describe the design of this model termed InterOMaX (INTERaction with Organoid-in-MatriX) as a bottom-up approach with the capacity for further complexity and as a bridge to 3D bioprinting technologies. While featuring key components of the TME, in vivo confirmation of the distinct T cell phenotype corroborates the physiological significance of this co-culture approach. The platform enables identification and validation of gene candidates for the T cell resistance phenotype in PDAC, with CXCL17 shown as an example to illustrate these capacities.

## Materials and methods

### Cell lines

Clonal cells originate from the primary heterogeneous cell line KPC7598 generated from a KPC PDAC tumor (B6.129S2-*Trp53*^*tm1Tyj/J*^ (Jackson #002109), *LSL-Kras*^*G12D*/+27^ and p48^*Cre/*+^ mice)^21^ through limited dilution. All KPC cell lines were maintained in DMEM (#10564011, Gibco) supplemented with 10% FBS and 100 units/mL penicillin/streptomycin. All cell lines were tested ‘free of mycoplasma’ (PCR Mycoplasma Test Kit; #A3744, AppliChem).

### Stable knock down of CXCL17 in KPC PDAC cells

Stable knock down of CXCL17 expression in resistant KPC PDAC cells was performed as described previously^22^. Five different vectors (Mission shRNA Plasmid DNA pLKO.1; TRCN0000180833, TRCN0000184757, TRCN0000196049, TRCN0000250216, TRCN0000250217) from Sigma, coding for shRNA against CXCL17 were tested. After selection with puromycin (2 μg/ml) the knock down cell line with the most reduced CXCL17 expression (Clone ID TRCN0000180833) was selected. Cells stably expressing scrambled shRNA served as control.

### Patient-derived organoids and isolation of human T cells from peripheral blood

Patient derived organoids (PDOs) were acquired from the Ulm University Organoid Core Facility. Generation and culture of PDOs were performed according to standardized protocols^23^. Prior to co-cultures, PDOs were cultured for at least 7 days in Matrigel domes with human complete feeding medium (hCPLT). hCPLT was prepared according to Boj et al.^24^ with minor adjustments (Supplementary Table S1). Peripheral blood buffy coats for T cell isolation were acquired from the blood bank of Ulm University Hospital from healthy donors. Isolation of peripheral blood mononuclear cells (PBMCs) and T cells was performed on the day before co-culture initiation. Co-cultures with human T cells were maintained in RPMI (#11875093, Gibco) supplemented with 10%FBS and 100 units/mL penicillin/streptomycin. PDOs and peripheral blood buffy coats were obtained after informed consent and approval by the Institutional Review Board (project numbers 72/19 and 105/23). All methods were carried out in accordance with relevant guidelines and regulations. Informed consent was obtained from all subjects and/or their legal guardian(s). Buffy coats were handled under S2 safety conditions. The blood was 1:1 diluted in DPBS (#14190144, Gibco) and a maximum of 35 ml of the dilution was gently transferred to 20 ml Ficoll-Paque™ PLUS (#17144002, cytiava). Centrifugation at 760 × g at room temperature for 20 min without brakes allowed for the separation of PBMCs from other blood components by density gradient. PBMCs were collected and washed in 15 ml DPBS with centrifugation for 8 min at 4 °C at 350 × g, respectively. For removal of platelets, the last washing step was performed with 10 min 200 × g centrifugation at room temperature. PBMCs were resuspended in DPBS with 2% FBS. T cells were isolated from PBMCs via negative selection by using magnetic activated cell sorting (MACS) with the Dynabeads™ Untouched™ Human T Cells Kit (#11344D, Invitrogen) according to the manufacturers’ instructions. In brief, superparamagnetic beads (4.0 µm diameter) were coated with a secondary polyclonal antibody that binds mouse IgGs. The antibody mix contains a cocktail of mouse IgGs that bind all immune cell markers which are not found on T cells. After adding the antibody mix to the sample to bind non-T cells, the beads were then added. After a short incubation, the bead-bound cells were separated using a magnet. The purified T cells in the supernatant were transferred for subsequent co-culture assays.

### Animal models

Mice were bred and kept under standard pathogen-free conditions in the animal colony of Ulm University (Ulm, Germany). All methods were carried out in accordance with relevant guidelines and regulations (Guide for the Care and Use of Laboratory Animals of the German Federal Animal Protection Law) and are reported in accordance with ARRIVE guidelines for the reporting of animal experiments. The protocols were approved by the Committee on the Ethics of Animal Experiments of the University of Ulm (Tierforschungszentrum Ulm, Oberberghof) and the Regierungspräsidium Tübingen (permit number 1589). 8-weeks old male C57BL/6 wildtype mice were utilized to establish allograft tumors subcutaneously in both flanks and for isolation of T cells from naïve mice. Immunizations were performed under short-time Isofluran anesthesia, and all efforts were made to minimize suffering.

### Isolation of T cells from mice and T cell activation

T cell isolation from tumor draining lymph nodes (TdLNs) or spleen was performed as described by Lin et al. 2022 (in vivo educated T cells; edT)^3^. Murine T cells were isolated via negative selection by using Dynabeads™ Untouched™ Mouse T Cells Kit (# 11413D, Invitrogen) according to the manufacturers’ instructions. For activation of T cells, cell culture plates were coated with an anti-Cd3e antibody (human: #555329, murine: #567115, BD) at 5 µg/ml and an anti-Cd28 antibody (human: #555725, murine: (#567110, BD) at 2.5 µg/ml 1 day prior to T cell isolation (in vitro plate coat-activated T cells; Tp).

### 2D T cell co-culture with KPC PDAC cells

T cells for co-culture assays were freshly isolated from C57BL/6 mice for co-culture. Co-cultures including T cells were maintained in RPMI (#11875093; Gibco) supplemented with 10% fetal bovine serum and 100 units/mL penicillin/streptomycin. 2000 KPC PDAC cells were seeded per well of a 96-well plate until becoming adherent. The medium was removed, and T cells were added to the co-culture at a ratio of 100:1 with 5 ng/ml murine IL-2 (#550069, BD). After 72 h endpoint analysis was performed.

### 3D T cell co-culture with KPC PDAC spheroids

**Figure 1:**
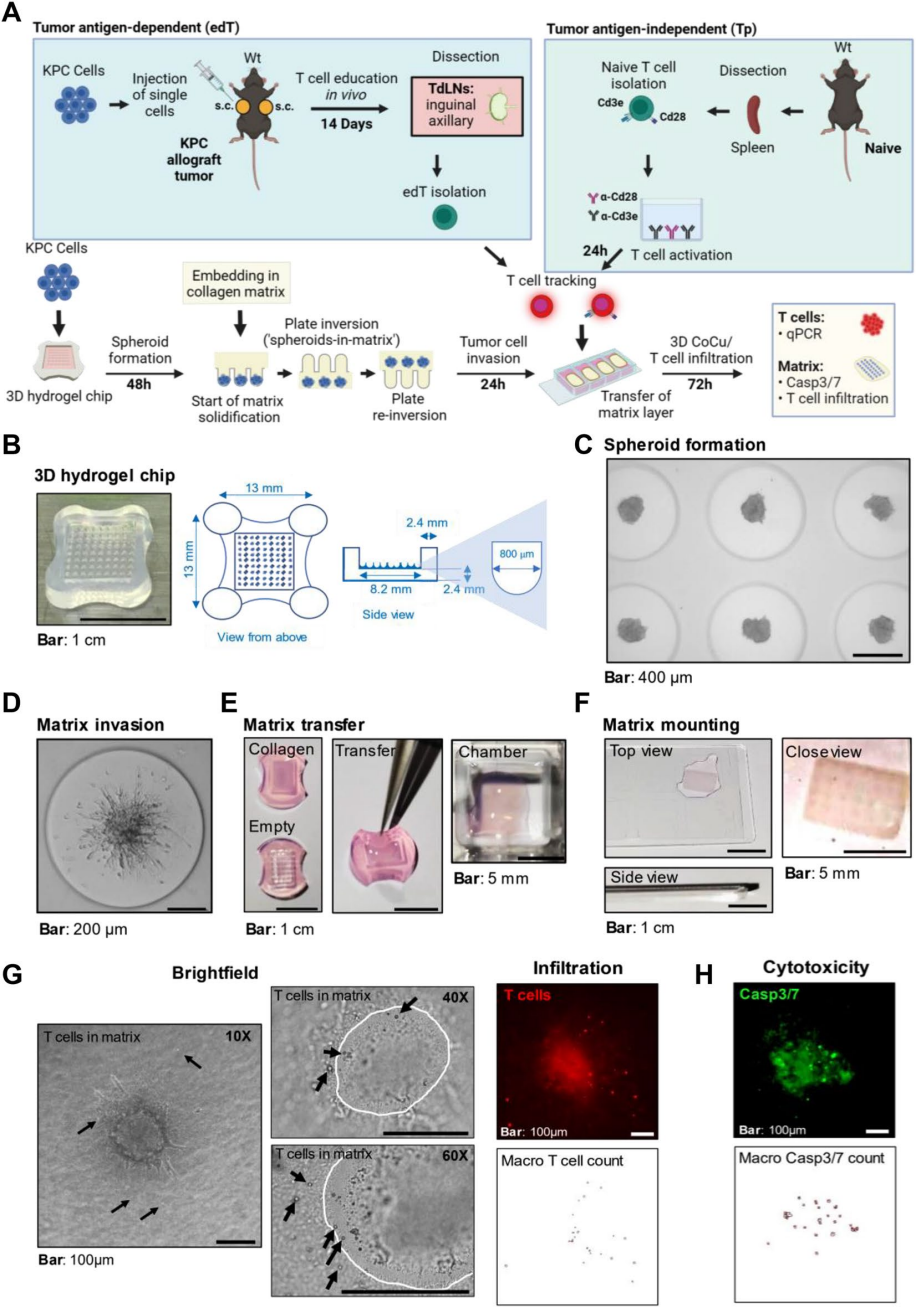
3D co-culture model system. (**A**) Overview and workflow. T cell interactions with KPC PDAC cells are analyzed using tumor antigen-independent (green box) or -dependent mechanisms (blue box). Tp: Tumor naïve splenic T cells activated via plate-coated Cd3e/Cd28 antibodies. EdT: Tumor-educated T cells from tumor-draining lymph nodes (TdLNs) of subcutaneous (s.c.) allograft tumor-bearing mice. Wt: wild-type mice. (**B**) Dimensions of an 81-microwell 3D agarose chip. (**C**) Representative brightfield (BF) image (4 × magnification) of KPC PDAC cells grown as spheroids for 48 h. (**D**) Representative BF image (10 × magnification) of a KPC PDAC spheroid grown for 48 h and embedded in 3 mg/ml collagen type I matrix for 24 h. (**E**) 3D agarose chips with or without collagen matrix (left) and the matrix patch transfer (middle) to chamber slides (right) using a fine-point tweezer. (**F**) Mounting of matrix patches onto chamber slides as top, side, and close-up view. (**G**) Representative fluorescence and BF microscopy images (10×, 40×, and 60× magnification) of 72 h infiltrated edT cells (indicated by black arrows). The spheroid border is outlined by a white dashed line. Right panel: Infiltrated edT cells into a spheroid previously stained with CellTrackerTM Deep Red and the corresponding segmentation image after application of the Fiji Macro for counting T cells. (**H**) Representative fluorescence image (10 × magnification) of Casp3/7 positive (apoptotic) KPC PDAC cells after 72 h of 3D CoCu with edT cells. KPC PDAC spheroids were stained with CellEventTM Caspase3/7 Detection Reagent prior to mounting of matrix patches onto slides. Below is the segmentation image for counting Casp3/7 positive cells using the Fiji Macro.

3D spheroid co-culture was performed as described in Lin et al.^3,16^. Briefly, 2% agarose chips were created using 35-well or 81-well casts from Microtissues^®^ (#A6013, Sigma-Aldrich). Matrix embedding with neutralized type 1 collagen (#08-115, Merck) was performed upon spheroid formation. The “Flip chip-to-embed”-method is shown as a movie in Lin et al.^16^ (see also Fig. 1A) and was applied to completely embed the spheroids in collagen within the agarose chip*.* After 24 h, the matrix patch was transferred to a chamber slide. T cells were stained with 1 µM CellTracker™ Deep Red Dye (#C34565, ThermoFisher) in serum-free RPMI medium for 45 min at 37 °C, resuspended in T cell medium and added at 10:1 T cell to cancer cell ratio on top of the matrix in the chamber slide. 3D co-culture was performed for 72 h until endpoint analysis.

### 3D human T cell co-culture with PDOs in agarose hydrogel chips

The PDO lines were derived from two different PDAC patients. Single PDO cells were seeded at 10,000 cells per 35-microwell agarose chip in 75 µl PDO growth medium until organoid formation after 72 h. PDOs were then embedded in 3 mg/ml collagen type I matrix according to our co-culture workflow. After 24 h, the matrix patch was transferred to a chamber slide and CellTracker™ Deep Red Dye stained pre-activated Tp cells added at a 10:1 T cell to PDO cell ratio to the chamber slide on top of the PDO-containing matrix patch in T cell medium supplemented with 5 ng/ml human IL2 (#554630, BD).

### 3D human T cell co-culture with PDOs in Matrigel domes

PDOs were grown on hydrogel chips for 72 h as described previously before carefully removing fully grown PDOs from the hydrogel chip by flushing them out from the chip. PDOs were subjected to standard dome culture using 35 µl Matrigel-based domes in 24-well plates. After 24 h, CellTracker™ Deep Red Dye-stained pre-activated T cells were added at a 10:1 T cell to PDO cell ratio into the well containing dome-based PDO culture in T cell medium supplemented with 5 ng/ml human IL2 (#554630, BD).

### Multicellular co-culture

PDOs were seeded at 10,000 cells per 35-microwell agarose chip until organoid formation. Afterwards, PDOs were stained with 10 µM CellTracker™ CMTPX Dye (#C34552, ThermoFisher). After 48 h, medium was removed and primary hPSCs were stained with 10 µM CellTracker™ Blue CMAC Dye (#C2110; ThermoFisher) prior to co-culture with PDOs at a ratio of 1:1 (10,000 per chip). hPSCs^25^ were maintained in DMEM supplemented with 20% FBS and 100 units/mL penicillin/streptomycin. Co-culture was incubated for 15 min at 37 °C before 1 ml of PSC medium (DMEM 20% FBS and 100 units/ml penicillin/streptomycin) was carefully added to each well. After 24 h, co-culture was embedded with 3 mg/ml type 1 collagen. After further 24 h, human Tp cells were added on top of the matrix in the agarose chip at 20:1 Tp cell to PDO cell ratio. Endpoint analysis was performed after 72 h of triple co-culture.

### T cell infiltration and cancer cell apoptosis assay

Detection of CellTracker™ Deep Red Dye-stained T cell infiltration was performed via fluorescence microscopy (BZ-X Series, Keyence) at 650 nm (Cy5 channel). For the apoptosis assay, Casp3/7 and Annexin V were utilized. KPC spheroids or PDOs were stained with CellEvent™ Caspase3/7 Detection Reagent (#C10423, Invitrogen) according to the manufacturers’ instructions. In brief, 2 drops of the dye per 1 ml T cell medium was applied and incubated for 1 h at 37 °C before fixation of the matrix patch with PFA. Fluorescent microscopic detection was performed at 530 nm (GFP channel). All images were quantified by automated cell counting of fluorescence positive cells through the z-axis at the level of spheroids/PDOs by using a Fiji Macro code (Supplementary Fig. S8). In average, at least 10 spheroids/PDOs were quantified per condition. For 2D co-culture, at least six different areas within the same culture dish were counted and averaged. Annexin V PE (#88-8102-72, Invitrogen) staining was performed after cell trypsinization of 2D grown KPC cells and used according to the manufacturer’s recommendation.

### Western blot

The preparation of cell lysates and its analysis by Western blotting was performed as described previously^22^. In brief, equal amounts of protein (24 μg) were loaded on the polymerized gel, which was blotted onto methanol activated PVDF membrane (GE Healthcare #10600021) for 18 h. The membranes were blocked with BSA containing blocking solution (5% BSA in TBST; BSA from Sigma-Aldrich #A9418) for 6 h (on orbital shaker at 20 rpm, 4 °C). Subsequently, membranes were incubated with primary antibodies diluted in blocking solution (1:10,000 anti-Gapdh Thermo #MA5-24222; 1:125 anti-Cxcl17 Thermo #MA524222) for 24 h ON (rolling, 4 °C). Visualization of protein bands was carried out according to the manufacturer’s instructions supplied with the Chemiluminescence HRP Substrate kit (Immobilon #WBKLS0500) and by using the chemiluminescence imager (FusionL Vilber lourmat). Analysis: Protein band intensities were quantified using the BandPeakQuantification Macro in Fiji (Supplementary Fig. S9). Intensity ratios (IR) were calculated by normalizing to GAPDH (loading control and housekeeping protein) protein band intensities. Fold change data were generated from IR data. Molecular weights were confirmed using colorimetrically visualized PageRuler™ Prestained Proteinladder.

### RNA extraction, quantitative RT-PCR and RNA sequencing

RNA extraction of KPC spheroids is described in Lin et al.^16^. The total RNA was extracted using RNeasy Mini kit (#74104, Qiagen) with on-column DNAse digestion. For qRT-PCR, reverse transcription was done using iScript cDNA Synthesis Kit (#1708890, Bio-Rad) according to the manufacturer’s protocol. qRT-PCR was performed with PowerUP™ SYBR™ Green Master Mix (#A25742, Applied Biosystems™). Primers were purchased from Qiagen (QuantiTect Primer Assay, #24990), and their sequences are provided in Supplementary Table S2. Fold change was calculated by 2^−ΔΔCT^ and relative expression by 2^−ΔCT^ normalized to *GAPDH*. All qRT-PCR assays were done in duplicates. For RNA-sequencing, samples were sent as triplicates to the Biomedical Sequencing Facility (BSF) of the CeMM Research Center for Molecular Medicine in Vienna. Primary analysis of BAM files was done by the BSF core facility in Vienna by use of the DEseq2 Bioconductor package. Resulting differential expression tables quantifying gene expression in counts per million and P value or comparisons were used to create a rank ordered list, which was then analyzed by the GSEA software provided by the Broad Institute in California (GSEA_4.3.2). The analyzed gene set originated from the Gene ontology (GO) database from the National Human Genome Research Institute. Rows were scaled by unit variance and the PCA method was SVD with imputation.

### Immunohistochemistry

CD3 immunohistochemistry staining was performed for paraffin sections from tumors dissected from mice 14 days after subcutaneous flank injection of T cell-resistant or -sensitive KPC PDAC cells. First, sections were deparaffinized using xylene (#1917524, Fisher Chemical) and hydrated using a standard alcohol (ethanol) series. After washing with ddH2O, antigens were retrieved using pre-warmed antigen unmasking solution (#H-3300, Vector) in a steamer for 35 min. Slides were washed in dH2O (2 × 5 min) and TBS-T (1 × 5 min), and endogenous peroxidase was blocked with 3% hydrogen peroxide solution (#1.07209.0250, Supelco) for 10 min at room temperature. After another washing step in dH2O and TBST, nonspecific antigens were blocked with 10% normal goat serum (#5425S, Cell Signaling) in TBST for 30 min at room temperature, and the Avidin Biotin Blocking Kit (#SP-2001, Vector) was used according to the manufacturer’s instructions. The primary antibody against mouse Cd3 (#ab16669, Abcam) was added at a 1:200 dilution in a wet chamber overnight at 4 °C. Slides were then washed in TBST (3 × 5 min), and the anti-rabbit biotinylated secondary antibody (#BA-1000–1.5, Vector) was applied at a 1:100 dilution for 45 min at room temperature. For color development, the VECTASTAIN^®^ ABC Peroxidase Kit (#PK-6100, Vector) was used according to the manufacturer’s instructions. After washing the slides in TBST, the signal was detected by using 10:1 diluted DAB HRP substrate (#11718096001, Roche) for 10 min at room temperature or until color development. Slides were counterstained using 20% hematoxylin (#1.0924, Merck) for 1 min and washed under tap water for color development. Slides were dehydrated using standard ethanol and xylene series prior to embedding in Entellan (#1.07961.0500, Merck). Samples with the secondary antibody only were used as negative control, and mouse spleen samples were used as positive control. By using the microscopy (BZ-X Series, Keyence), ten brightfield images (10× magnification) per condition were taken and automatically analyzed using the Fiji Macro (Supplementary Fig. S10). The Macro counts both Cd3 positive cells and all cells, allowing for the calculation of the percentage of Cd3 positive cells per area. Representative Images were taken at 20× magnification.

### Immunofluorescence

Immunofluorescence was used for the detection of the standard patient-derived organoid (PDO) markers Ck19, Ki67, and E-Cadherin. 10,000 PDO cells were seeded into 35-microwell hydrogel chips and grown for 72 h in PDO growth medium before embedding in a 3 mg/ml collagen type I matrix ("organoids-in-matrix", Fig. 3A). After 24 h, the medium was changed to T cell medium, and PDOs were cultured in the matrix for another 72 h. Subsequently, matrix patches were transferred to chamber slides using a fine-point tweezer, and patches were washed with DPBS. PDOs were fixed using 4% PFA (#158127, Sigma-Aldrich) for 1.5 h at room temperature. After another washing step in DPBS, nonspecific antigens were blocked using 2.5% BSA (#A9418, Sigma-Aldrich) in DPBS for 20 min at room temperature. Primary fluorophore-conjugated antibodies for staining Ck19 (AF546; sc-6278, Santa Cruz, 1:100 dilution), Ki67 (AF488; sc-23900, Santa Cruz, 1:50 dilution), and E-Cadherin (primary antibody: #3195S, Cell Signaling, 1:100 dilution; secondary antibody: anti rabbit AF568; #A10042, Invitrogen) were added in 1.25% BSA/DPBS overnight at 4 °C. Matrix patches were washed in DPBS (3 × 10 min at the rocker) prior to staining with 1:2000 diluted DAPI solution (#1351303, Bio-Rad) for 3 min at room temperature. Patches were then washed 3 × in DPBS and embedded using Fluoromount-G™ Mounting Medium (#00-4958-02, ThermoFisher). Overview fluorescence images at 4× magnification were taken using the BZ-X Series Keyence microscope (AF546—TRITC channel; AF488—GFP channel; AF647—Cy5 channel).

### Confocal images

Confocal images were acquired using a Leica TSC SP8-HCS system with a HC PL APO 20x/0.75 IMM CORR CS2 objective and HyD detector. For 3D renders of organoids, system-optimized airy z-stacks were acquired and subsequently combined using the Leica 3D rendering software module. Single z-section merges display subcellular localization of markers.

### Statistical analysis

All statistical tests and graphing were carried out using GraphPad Prism 9.5.1, Microsoft Excel, ClustVis (https://academic.oup.com/nar/article/43/W1/W566/2467929), Kaplan–Meier plotter (https://pubmed.ncbi.nlm.nih.gov/36856946/), TIMER2.0 (https://pubmed.ncbi.nlm.nih.gov/32442275/), and GSEA_4.3.2 (https://www.pnas.org/doi/10.1073/pnas.0506580102, https://www.nature.com/articles/ng1180). Unpaired t-test was used for dichotomous comparisons. Multiple unpaired t-tests or two-way ANOVA were used for multiple comparisons with P < 0.05 (dichotomous) or P-adjusted < 0.05 (multiple testing) as the threshold for statistical significance in all tests.

## Results

### Advancement of a 3D co-culture setup to a more versatile model system

We designed a cancer cell/T cell 3D co-culture assay to more closely mimic the TME based on a previously established model for PDAC^16^. First, a collagen matrix in which the cancer cell spheroids are completely embedded within an agarose microwell is generated. Added T cells can then infiltrate from the outside of the matrix in the subsequent co-culture assays reflecting barriers in the TME T cells need to overcome. To uncover a tumor antigen-specific T cell response to co-cultured PDAC spheroids, we utilized tumor-educated (ed)T cells isolated from inguinal and axillary tumor-draining lymph nodes (TdLNs) of immunocompetent C57BL/6 mice carrying subcutaneous PDAC allografts grown for 14 days. For generic T cell interaction, splenic T cells from tumor-naïve mice were activated with plate-bound anti-Cd3e/Cd28-antibodies (Tp cells) to serve as controls for tumor-antigen dependent T cell responses. After co-culture, we performed qPCR of effector T cell activation markers (IL-2, GZMB, TNFA, IFNG, EOMES) and FOXP3 for regulatory T cells, immunofluorescent microscopy to quantify Casp3/7 positive (apoptotic) PDAC cells and CellTracker™ Deep Red stained T cell infiltration, as well as transcriptomic analysis of PDAC cells in the matrix (Fig. 1A). We collected the T cell fraction from the cell culture medium outside the matrix for qPCR analysis of immune-related genes, therefore no cell separation from the matrix was required. Due to paracrine crosstalk between PDAC cells with T cells through the matrix, the expression data of T cells outside the matrix is representative for the underlying PDAC immune-phenotype. The multiple readouts resulting from the co-culture studies provide orthogonal readouts of the T cell response phenotype of PDAC cells. Agarose multi-microwell chips^3,16^ were used to generate and seed multiple PDAC spheroids in a single pipetting step (Fig. 1B). Due to the small size of the microwells (800 μm in diameter), cell–cell interaction is maximized, resulting in a rapid generation of uniformly-sized 3D cultures within one agarose chip (Fig. 1B,C). Upon matrix-embedding, cancer cells invade the surrounding collagen matrix (Fig. 1D). The “spheroids-in-matrix patch” can be completely separated from the agarose array and transferred to a well in a chamber slide for co-culture with T cells in suspension (Fig. 1E). After co-culture, matrix patches can be stained (Fig. 1E), transferred to a glass slide, mounted (Fig. 1F) and subjected to immunofluorescent microscopy (Fig. 1G,H) for analyzing T cell infiltration (Fig. 1G, far right) and T cell-mediated cancer cell apoptosis (Fig. 1H). Using the ImageJ Macro Watershed Segmentation technique, automated and standardized counting of infiltrated T cells was applied (Fig. 1G,H, Supplementary Fig. S8). During the establishment phase, we compared automated with manual counting results for validation. The results were highly comparable between both methods (data not shown).

In summary, we established a 3D co-culture platform that mimics the TME to investigate parameters of T cell sensitive and resistant phenotypes of PDAC cells.

### Uncovering distinct T cell response phenotypes of KPC PDAC cells with corresponding transcriptomic profiles

**Figure 2:**
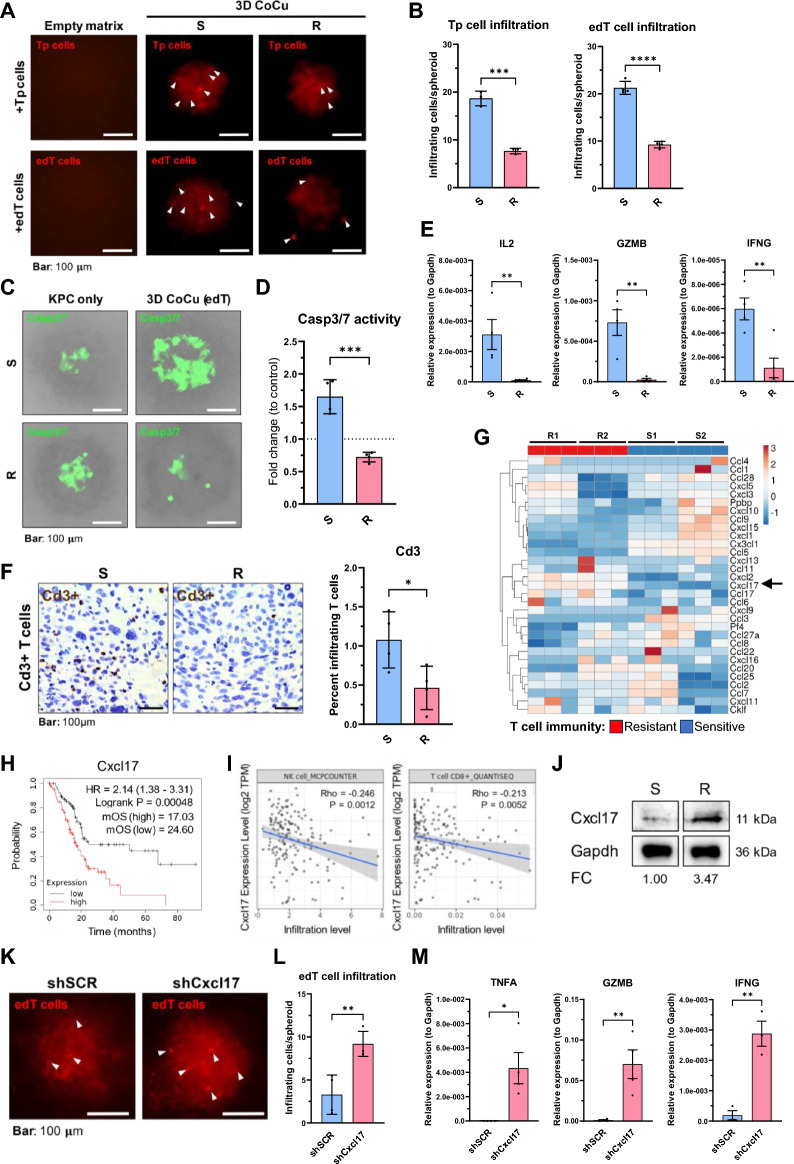
Identification of distinct KPC PDAC T cell response phenotypes. (**A**) Representative fluorescence images showing Cd3e/Cd28 plate-activated T (Tp) cells or tumor-educated (edT) T cells infiltrating into collagen I matrix with resistant (R) or sensitive (S) KPC spheroids after co-culture (CoCu) for 72 h. Detection of CellTrackerTM Deep Red-stained as indicated by white arrows. (**B**) Number of infiltrated T cells/spheroid counted with Fiji fluorescence Macro. Means were calculated for 10 spheroids/condition. N ≥ 3. (**C**) Representative fluorescence images showing Casp3/7 positive R- or S-cells 72 h after Co-culture with edT cells. (**D**) Number of Casp3/7-positive cells/spheroid counted with Fiji Macro. Means were calculated for 10 spheroids/condition. N = 4. (**E**) Relative expression (2^−∆Ct^) via RT-qPCR of T cell activation markers in edT cells after 3D CoCu with R- or S-spheroids. N ≥ 4. (**F**) Infiltration of Cd3 positive T cells into s.c. allograft tumors from S- and R-cells. Percentage of infiltrating T cells in relation to all cells was detected by DAB staining immunohistochemistry and quantified using the Fiji DAB Macro in 10 areas/condition. N = 4. (**G**) Heatmap for all chemokines from the mouse GO chemokine activity pathway showing RNAseq expression data of two resistant (R1, R2) and two sensitive (S1, S2) clonal KPC cell lines. CXCL17 is highlighted with an arrow. N = 3. (**H**) High CXCL17 levels indicate a worse prognosis in patients with PDAC. KM plot showing progression free survival in patients with stratified CXCL17 expression. n = 177 patients. (**I**) High CXCL17 expression is correlated with decreased Cd8 + T cell and Nk cell infiltration. TIMER2.0 showing correlation of CXCL17 expression in the PDAC dataset with gene signatures for Cd8 + and Nk cells. N = 177. (**J**) Representative protein expression for Cxcl17 from S- or R-cells. Band densities were normalized to Gapdh and are shown as R/S fold change. (**K**) Representative fluorescence images showing edT cells infiltration into shSCR or shCxcl17 KPC spheroids after co-culture. Infiltrating T cells are indicated by white arrows. (**L**) Number of infiltrated T cells/spheroid. Means calculated for 8 spheroids/condition. N = 4. (**M**) Relative expression (2^−∆Ct^) via RT-qPCR of T cell activation markers in edT cells after co-culture with shSCR or shCxcl17 spheroids. Results were normalized to GAPDH. N ≥ 3 independent biological replicates. *p ≤ 0.05, **p ≤ 0.01, ***p ≤ 0.001, ****p ≤ 0.0001.

We utilized four clonal derivative cell lines from the primary heterogeneous KPC7598 PDAC cell line to examine distinct PDAC clone-dependent T cell responses in the readouts. Responses were compared for in vitro T cell infiltration (Fig. 2A,B), T cell-mediated cancer cell apoptosis (Fig. 2C,D), effector T cell activation markers (Fig. 2E), in vivo T cell infiltration (Fig. 2B,F), as well as by comparative transcriptomic analysis (Fig. 2G, Supplementary Fig. S1). Indeed, we identified PDAC clones with significantly decreased T cell infiltration and T cell-mediated PDAC cell apoptosis and grouped them into “T cell-resistant” (R) vs. “T cell-sensitive” (S) PDAC clones (Fig. 2A–D). We used an empty matrix (without embedded PDAC cells) as control to rule out random T cell infiltration (Fig. 2A). We found increased T cell infiltration upon co-culture with both edT cells and Tp cells, indicating that tumor-antigen independent mechanisms contribute to T cell-mediated cytotoxicity (Fig. 2A,B). Also, the expression of effector T cell activation markers (IL-2, GZMB, IFNG) in edT cells were significantly lower after co-culture with T cell-resistant (R) as compared to -sensitive (S) cancer cells (Fig. 2E), indicating decreased T cell effector functionality and thus impaired PDAC cell killing. However, the spheroid area did not differ between S- and R-spheroids in T cell co-culture (Supplementary Fig. S2). The physiological relevance of our 3D model was corroborated in animal studies where we observed significantly increased Cd3^+^ T cell infiltration into s.c. tumors of mice injected with sensitive compared to tumors from resistant cells plates (Fig. 2F, Supplementary Fig. S3). We also compared the 3D co-culture results with the ones generated from a standardized 2D co-culture setup in a parallel approach (Supplementary Fig. S4). Here, edT cells or Tp cells were directly co-cultured with adherent KPC PDAC cells in tissue culture (Supplementary Fig. S4A,B). We additionally utilized a flow cytometry-based Annexin V apoptosis assay as an independent assessment method for Tp cell-mediated cytotoxicity indicated by the Casp3/7 assay via immunofluorescence (Supplementary Fig. S4C–E). We found that PDAC cell apoptosis and expression of effector T cell activation markers were comparable between a standardized 2D co-culture and our 3D co-culture system. However, in comparison with a standardized 2D T cell co-culture setup, the main advantages of our 3D co-culture system are more physiological culture conditions, generation of multiple readouts, the possibilities to monitor T cell infiltration, and compartmentalization of different cell lines through a customizable matrix. To investigate gene expression and pathway enrichment regulating the T cell response, comparative transcriptomic analysis of S- and R-cells was performed. Here, we show the analyses of two representative S- and R-cell lines, respectively. Matching with the observed phenotypes, Gene Set Enrichment Analysis (GSEA) showed immunologically relevant pathways, such as *Positive regulation of T helper 1 type immune response, CD8 positive alpha beta T cell activation, MHC protein complex binding*, and *Positive regulation of chemokine production*, being significantly enriched in S- vs. R-cells (Supplementary Fig. S1). Since paracrine crosstalk through the matrix most likely contributed to the observed T cell response phenotype, we sought to analyze differentially expressed chemokines between S- and R-cells. All chemokines (GO chemokine activity pathway; MGI (mouse); GO008009) were selected from the RNAseq gene list and used for heatmap clustering of S- and R-cells, respectively (Fig. 2G). Some chemokines show upregulation in both S- compared to R-cell lines, e.g. CCL5 and CX3CL1. Accordingly, CCL5 (RANTES) has been shown to enhance recruitment of effector T cells^26,27^. Only the two chemokines CXCL17 and CXCL2 showed downregulated expression in both S- compared to R-cells. CXCL17 expression correlated with both worse survival (Fig. 2H) and reduced infiltration of CD8 + T cells and natural killer (NK) cells in patients with PDAC (Fig. 2I).

In summary, this 3D co-culture model system was highly reliable in distinguishing between a T cell-sensitive and -resistant KPC PDAC cell phenotype, which is also reflected in the transcriptomic profiling. This facilitates the identification of genes and pathways modulating the T cell response to PDAC. T cell infiltration into tumors of the allograft mouse model confirmed a higher number of intratumoral T cells in tumors derived from sensitive compared to resistant PDAC cells, which corroborates the physiological significance of the ex vivo co-culture approach. The multiple readouts complement each other and provide orthogonal indicators of the T cell response phenotype of the PDAC model cancer cells with a robust and versatile 3D phenotype-imaging-transcriptomic platform. From these analyses we identified CXCL17 as a potential candidate for T cell resistance in PDAC.

### CXCL17 contributes to the T cell resistant phenotype in KPC PDAC cells

Next, we sought to validate the impact of CXCL17 in mediating T cell resistance in our model system. First, we confirmed that R-cells showed higher CXCL17 expression at the protein level compared to S-cells (Fig. 2J). After shRNA-mediated knock down of CXCL17 of 79% (0.21-fold downregulated relative to control) in R-cells (Supplementary Fig. S5A), knock down cells showed an increased T cell infiltration rate (Fig. 2K,L), higher expression of T cell activation markers (Fig. 2M) as well as increased T cell-mediated cancer cell apoptosis (Supplementary Fig. S5B) compared to scrambled control cells.

In summary, knock down of CXCL17 in resistant cells caused T cell-sensitization and increased cancer cell cytotoxicity. The expression of CXCL17 is rate limiting for mediating T cell-resistance since its knock down resulted in T cell-sensitization. Thus, CXCL17 was validated as a rate limiting candidate for T cell resistance in our model system.

### A human PDAC organoid-in-matrix T cell co-culture platform reveals distinct T cell responses

**Figure 3:**
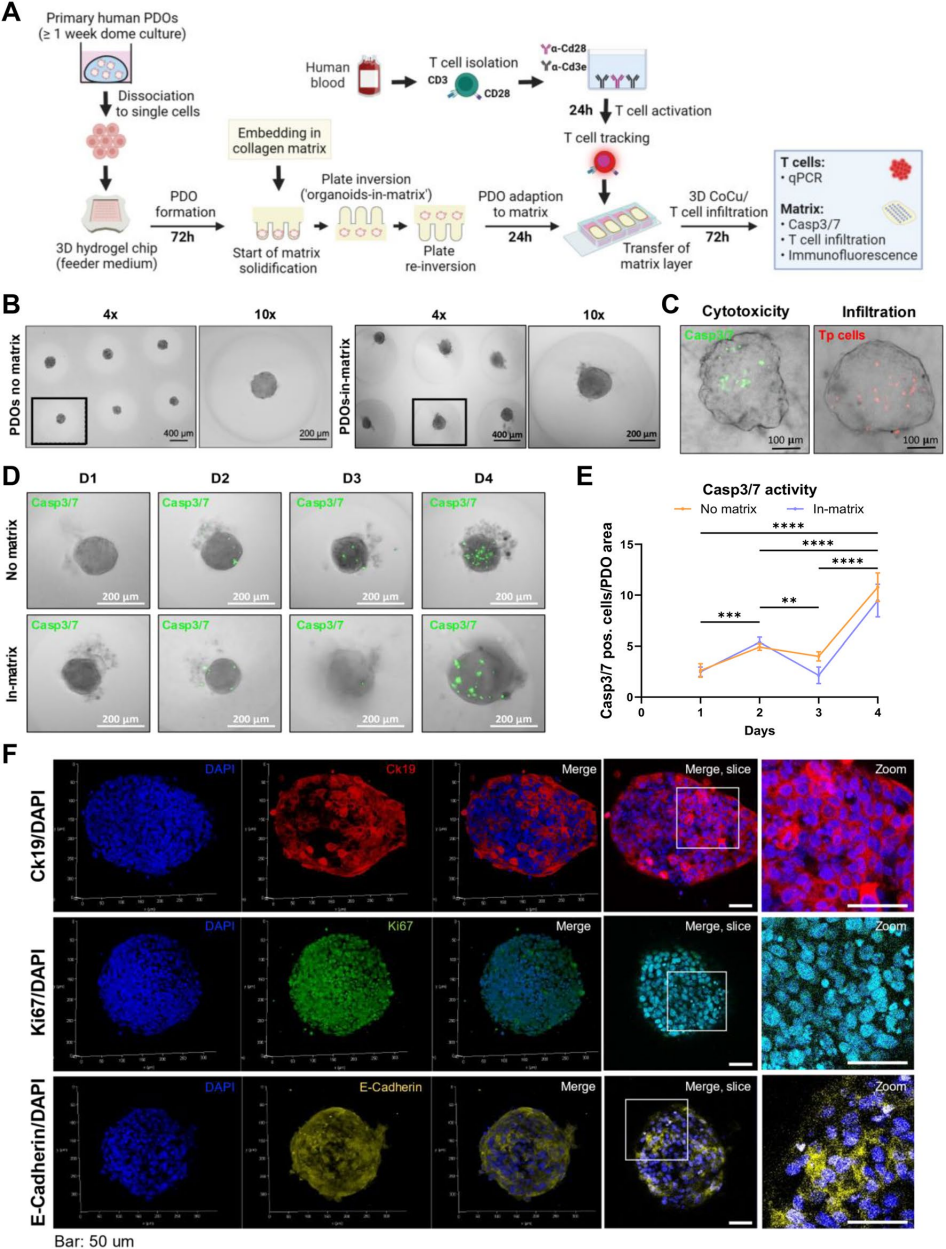
Integration of the patient-derived organoid (PDO) platform into the 3D co-culture model and development of the InterOMaX (INTERaction with Organoid-in-MatriX) platform. (**A**) Schematic overview of the workflow. PDOs are formed for 72 h in 3D agarose chips, then embedded in 3 mg/ml collagen I matrix. Following 24 h of adaption to the matrix, matrix patches are transferred to chamber slides. CellTrackerTM Deep Red-stained human Tp cells are added and co-cultured (CoCu) for 72 h. Tp: Pre-activated T cells with plate-coated Cd3e/Cd28 antibodies isolated from PBMCs of buffy coats from healthy donors. (**B**) Representative brightfield (BF) images (4× or 10× magnification) of PDOs in hydrogel chips, either grown for 72 h without matrix (left panel) or grown for 96 h with collagen matrix embedding after 72 h (right panel). (**C**) Merged BF and fluorescence microscopy images (10 × magnification) of infiltrating human Tp cells (right) or Casp3/7-positive PDO cells (left) after 72 h of 3D CoCu. Tp cells were stained with CellTrackerTM Deep Red. Apoptotic cells were stained using the CellEventTM Caspase-3/7 Detection Reagent. (**D**) Representative fluorescence microscopy images (10× magnification) of Casp3/7-positive PDO cells. PDOs were grown in T cell medium for four days (D1–D4), with or without collagen matrix embedding. Embedding was performed 48 h before D1 and feeder medium was changed to T cell medium 24 h before D1. Apoptotic cells were stained as indicated in (**C**). (**E**) Number of Casp3/7-positive cells per PDO in relation to PDO area over time. Means were calculated for ten PDOs, respectively. N = 3. (**F**) Representative confocal microscopy fluorescence images for immunofluorescence of indicated PDO markers. Markers are shown as 3D z-stack renders and merge of single Z-slice airy sections as well as respective zoom-in images. Scale: 50 µm. N ≥ 3 independent biological replicates.

Current generation of organoid “dome” cultures lack (i) a customizable matrix (ii) well-defined locations of the organoids within the matrix, and (iii) dynamic interaction with T cells. Therefore, we sought to integrate human organoids into the advanced 3D co-culture system that is based on a microarray setup and generated the InterOMaX (INTERaction with Organoid-in-MatriX) platform. Importantly, this platform addresses the major shortcomings of current patient-derived organoid (PDO) cultures. The workflow and readouts of the 3D human organoid-based co-culture (Fig. 3A) are similar to the murine spheroids shown above (Fig. 1A). The InterOMaX platform was established employing pre-activated T cells (Tp cells) from human peripheral blood mononuclear cells (PBMCs) of healthy donors by plate-coated anti-Cd3e/Cd28-antibodies. One of the important distinctions of InterOMaX from the classical dome culture is the initial seeding of a pre-defined number of PDO cells into the agarose microarray chip. This results in an equal PDO cell distribution in each microwell and therefore a more rapid and controlled generation of uniformly-sized PDOs in well-defined cell-to-cell distances (Fig. 3B, left). Analysis of T cell infiltration and T cell-mediated cancer cell apoptosis (Fig. 3C) was performed accordingly to the murine system (Fig. 1G,H). Importantly, in the InterOMaX PDOs are derived from a mix of single cells, thus maintaining the heterogeneity present in each organoid. Collagen embedding was performed after organoid formation in microwell casts and did not disturb the PDO structure or impact their distribution within the agarose chip (Fig. 3B, right). Furthermore, compared to the classical organoid dome culture, organoids of the InterOMaX platform are located in the same optical plane, which makes it amenable for reproducible and automated, high-throughput imaging. This is achieved by the previously described “flip chip-to-embed”-method, which requires stringently timed inversion steps of the agarose microwell chip adjusted to the matrix concentration and composition, as well as the size of the agarose chip^16^. The growth over time in the absence of matrix embedding resulted in a maximum size of PDO at around day 6–7 after cell seeding (Supplementary Fig. S6A,B). PDO growth rate in T cell medium over a time period of 4 days after cell seeding was comparable between experimental settings without or with embedding in a matrix (Fig. 3D, Supplementary Fig. S6C). Notably, spontaneous PDO apoptosis normalized to PDO area significantly increased at day 4 with or without matrix (Fig. 3D,E). With our co-culture workflow, PDOs are formed within 3 days and co-culture was performed 1 day after matrix embedding (Fig. 3A). This allowed for the maintenance of a relatively low spontaneous PDO apoptosis rate prior to T cell co-culture, providing a stable and low background signal for quantifying T cell-mediated apoptosis of PDOs. Human PDOs cultured in the agarose array system showed expression of the respective markers of PDAC including cytokeratin 19 (Ck19), Ki67 and Epithelial Cadherin (E-Cadherin) (Fig. 3F, Supplementary Fig. S7). Ck19 is a component of the intermediate filament network of epithelial cells, and had been shown to be located at the cell surface in PDAC. In accordance with the literature^28^, Ck19 is located in the cell periphery in our staining, rather than in the center of cells within the organoids. Ki67 is a proliferation marker that is highly expressed in differentiated, thus proliferative PDAC organoids^29^. Since Ki67 is associated with cell proliferation it is mostly detected in the nucleus. Therefore, it was expected to find high Ki67 + expressing PDOs, which mostly co-localized with the DAPI staining. These data show that T cell media neither inhibit PDO growth nor influence the maintenance of PDO marker expression (Fig. 3D–F).

**Figure 4:**
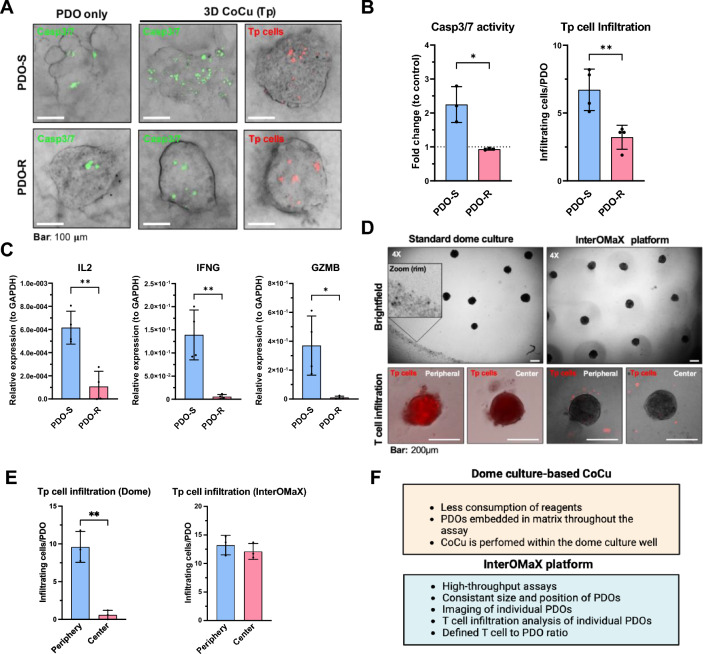
Identification of distinct T cell immune response phenotypes in human PDOs using the InterOMaX (INTERaction with Organoid-in-MatriX) platform. (**A**) Representative merged brightfield (BF) and fluorescence images for pre-characterized Tp cell response of sensitive and resistant PDO lines (PDO-S and PDO-R) from two different patient tissue samples. PDO culture without Tp cells was used as control for the Casp3/7 activity assay. Bars are set as indicated. (**B**) Number of fluorescence-positive cells counted per PDO through the z-axis analyzed by using the Fiji Macro. Means were calculated for ten PDOs per condition. Results for Casp3/7 are shown as fold change to control. N ≥ 3. (**C**) Relative expression (2^−∆Ct^) via RT-qPCR of given T cell activation markers in Tp cells after 72 h co-culture (CoCu) with PDO-R or PDO-S pre-characterized PDOs. Results were normalized to *GAPDH*. N = 4. (**D**) PDOs grown in hydrogel chips for 72 h were either subjected to 3D T cell CoCu using the InterOMaX platform or Matrigel dome-based cultures. Representative BF images (4× magnification) and merged BF/fluorescence images (10× magnification) show Tp cell infiltration for both models in peripheral and central regions, respectively. A zoomed-in view of the rim of the dome culture is shown in the left upper panel. Tp cells were stained with CellTracker™ Deep Red prior to start of 3D CoCu. Bars as indicated. (**E**) Number of infiltrated Tp cells into PDOs in dome culture and InterOMaX platform in peripheral and central areas, respectively. Tp cells were counted with Fiji fluorescence Macro. Quantification ≥ 3 PDOs from different areas per condition. N = 3 (**F**) Comparison of advantages between the dome culture-based and InterOMaX 3D CoCu platforms. N ≥ 3 independent biological replicates. *p ≤ 0.05, **p ≤ 0.01.

Similar to the above KPC PDAC spheroid co-cultures, T cell infiltration and T cell-mediated PDO cytotoxicity can also be measured and quantified in an automated manner (Figs. 3C, 4A–C). In a side-by-side comparison between Matrigel dome culture-based co-culture and the InterOMaX platform, we found that analysis and quantification of T cell infiltration into each individual organoid is more feasible and consistent in our platform (Fig. 4D–F). For starters, the distribution of the PDOs in the microarray setting is more uniform with well-defined distances from each other, whereas PDOs in the Matrigel domes are distributed at random. To create comparable co-culture conditions with equally-sized PDOs, we generated the same number of PDOs for both InterOMaX and dome-based co-cultures in the microarrays and transferred the PDOs into dome cultures afterwards. Due to the given shape of the dome, T cells accumulated at the rim of the dome where they started to infiltrate, but barely reach PDOs located in the center of the dome (Fig. 4D). In contrast, due to the thin and flat shape of the matrix in the InterOMaX platform, all PDOs were equally exposed to T cells from outside the matrix, resulting in comparable T cell infiltration rates across all PDOs in the matrix (Fig. 4D,E). By employing the InterOMaX platform, we distinguished T cell-sensitive (S) and -resistant (R) PDOs, reflected in their distinct T cell infiltration and PDO apoptosis after Tp cell co-culture (Fig. 4A–C). T cell activation marker (IL-2, IFNG, GZMB) expression in Tp cells was significantly lower after co-culture with PDO-R compared to PDO-S (Fig. 4C). Thus, we were able to combine the advantages of both organoid culture and the InterOMaX platform, capturing tissue complexity and well-defined, tailored experimental conditions for probing dynamic cellular behavior critical for drug and T cell therapy response in PDAC.

### Multicellular dynamic interactions contribute to T cell functionality

**Figure 5:**
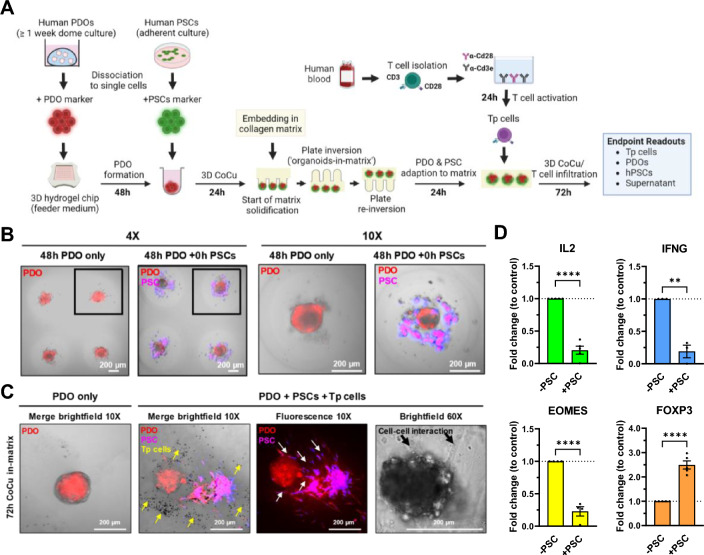
Impact of multicellular co-culture of human PDOs and PSCs on T cell functionality. (**A**) Schematic overview of the workflow. PDOs were stained with CellTacker™ Red and cultured in 3D hydrogel chips for 48 h prior to adding CellTacker™ Blue-stained hPSCs for further 24 h. Afterwards, cells were embedded in collagen type I (3 mg/ml) matrix. After 24 h, human peripheral, pre-activated T cells (Tp) were added from outside the matrix for 72 h. Tp cells were pre-activated with plate-coated Cd3e/Cd28 antibodies after isolation from PBMCs of buffy coats from healthy donors. (**B**) Representative merged brightfield (BF) and fluorescence images (4× or 10× magnification) of PDOs and hPSCs in 3D hydrogel chips. Images were taken after 48 h of PDO culture or after adding hPSCs to 48 h cultured PDOs. (**C**) Representative merged brightfield and fluorescence or fluorescence only images (10× or 60× magnification) of PDOs, hPSCs, and Tp cells in 72 h (from the start of triple co-culture) matrix-embedded 3D hydrogel chips. PDOs and hPSCs were stained as described in (**B**). Images show PDO only (left), PDO with hPSCs and T cells in 3D triple co-culture (middle), and PDO with PSC cell–cell interaction at 60× magnification (right). Yellow arrows indicate Tp cells. White or black arrows indicate hPSCs protrusions growing towards the PDO. (**D**) Fold change relative to control (2^−ΔΔCt^) expression via RT-qPCR of the given T cell markers in human Tp cells after 72 h of co-culture in 3 mg/ml collagen matrix with PDOs and ± hPSCs. Tp cells in co-culture with PDOs and without hPSCs served as control. Results were normalized to *GAPDH*. N ≥ 3 independent biological replicates. Significance **p ≤ 0.01, ****p ≤ 0.0001.

To approximate recreation of cellular key components of the in vivo TME in the InterOMaX platform, we sought to include primary human pancreatic stellate cells (hPSCs) into the PDO–T cell co-culture to investigate the impact on T cell effector functionality. First, we employed direct co-culture of PDOs and hPSCs upon organoid formation (Fig. 5A). After matrix embedding of PDOs, human Tp cells were added from the outside of the matrix to mimic the previous workflow (Fig. 5A,B). The majority of matrix-infiltrating Tp cells were located in the surrounding area of the PDOs, but did not infiltrate into the PDOs (Fig. 5C). In the presence of hPSCs, the expression of T cell activation markers IL-2, IFNG, EOMES was decreased and the expression of the regulatory T cell marker FOXP3 was increased (Fig. 5D). This indicates an hPSC-mediated inhibition of cytotoxic T cell functionality. T cell infiltration was not assessed in these studies, instead the expression of the above-mentioned T cell activation markers was used as a representative readout for T cell responses in co-culture. If needed, quantification of infiltrated T cells is feasible by pre-staining the different cells with different dyes.

In conclusion, the presence of hPSCs impairs effector T cell functionality towards PDO cytotoxicity and shows the applicability of the InterOMaX platform for recreating the complex PDAC TME with patient-matched key components of the tumor niche.

## Discussion

Here, we present a refined and reproducible 3D co-culture-in-matrix-based imaging-transcriptomic platform for probing cellular crosstalk to uncover underlying gene and pathway signatures associated with T cell response. This versatile co-culture platform enables reliable differentiation between distinct T cell response phenotypes shown here for PDAC subpopulations by generating complementary readouts of the T cell response phenotype. Compared to standard 2D and 3D dome culture-based co-culture systems, the major benefits of this platform are (i) high-throughput assay format with uniformly sized and well-controlled organoid positioning through microarray setup, (ii) maintenance of heterogeneity within the organoids, (iii) more accurate quantification and analysis of T cell infiltration into homogeneously shaped individual organoids, and (iv) potential for multiple complementary readouts. Notably, we confirmed distinct T cell infiltration rates into T cell-sensitive and -resistant PDAC cells in animal allograft studies of the cancer cells, thus demonstrating the physiological significance of the ex vivo co-culture approach (Fig. 2F). Moreover, this co-culture platform supports the identification and validation of gene candidates for T cell resistance (Fig. 2G–M, Supplementary Fig. S5), exemplified by our findings for CXCL17. CXCL17 is known to accelerate tumor progression, and has been described as an immunosuppressive chemokine in hepatocellular carcinoma^30^. However, its role in PDAC appears contradictory: Hiraoka et al. suggested that CXCL17 might be involved in anti-tumor immune response during pancreatic carcinogenesis. In premalignant intraductal papillary mucinous neoplasm, CXCL17 induced DC accumulation at the tumor site, which promoted tumor cell susceptibility to cytotoxic T cell-mediated cytolysis^31^. In contrast, CXCL17 expression is negatively correlated with patients’ survival as well as CD8^+^ T cell and NK cell infiltration (Fig. 2H,I). The platform shown here holds the potential to identify and elucidate the role of less known gene candidates in cancer cell-mediated T cell response.

The additions to our first-generation 3D co-culture model system^16^ enable minute cancer cell-T cell and -stromal cell interactions through a customizable matrix. Since there is no “one-fits-all” matrix composition for PDAC, this enables an effective control and malleability of the noncellular PDAC microenvironment. Furthermore, we deployed the InterOMaX platform to study the interplay of different cell populations and thus aid in understanding cellular behavior and how it relates to function. We first established and validated this system with murine KPC clonal PDAC spheroids, and now also integrated patient-derived PDAC organoids into the system. The incorporation of human organoids into our model system further permitted the combination of tissue complexity with well-defined, highly reproducible and high-throughput assay performance. While PDOs in dome cultures originate from single cells, PDOs generated in our model are generated from a cell mixture, thus preserving cancer cell heterogeneity within each organoid. One of the major challenges in culturing PDOs over time is to control cell death in the core of the growing organoids. In our model, spontaneous PDO apoptosis rate was negligible until day 3 after cell seeding for both matrix and non-matrix culture conditions. T cell co-cultures start within this time frame of a low background apoptosis rate.

The murine gene expression data from sensitive and resistant cancer cells were derived from clonal cell lines. Thus, a direct comparison with gene expression data from the heterogeneous PDOs to identify candidates of T cell resistance may be biased by the cellular composition of these PDOs. For future studies, employing comparative transcriptomic data from resistant and sensitive clonal PDOs would be a more relevant approach for comparison with expression data from our clonal KPC cells.

One of the features of the platform is the utilization of tumor antigen-specific T cells harvested from draining lymph nodes in tumor-bearing immunocompetent mice. Previous studies used non-specifically activated T cells or genetically engineered T cells for co-culture experiments^25,32^. We generated tumor-educated, antigen-specific T cells and compared the effector T cell functionality effects on PDAC cells between tumor-educated and anti-Cd3e/Cd28-activated T cells. This approach enabled a better differentiation between tumor antigen-dependent versus -independent T cell response mechanisms, e.g. soluble factor-mediated T cell immunomodulation. This differentiation would not be possible in co-culture models where T cells were designed to recognize common cell surface peptides^32^. In our murine co-culture studies, edT cell and Tp cell effects on T cell infiltration and PDAC cell apoptosis were comparable (Fig. 2B–E, Supplementary Fig. S2C–F), indicating that tumor antigen-independent mechanisms contribute to modulate a T cell response in these PDAC cells. We utilized T cells from axillary and inguinal tumor-draining lymph nodes (TdLNs), which are known sources for tumor-reactive T cells of the murine allograft model^16^ and showed a distinct cellular composition between immunotherapy responders and non-responders in previous studies^16^. Moreover, a substantially higher number of vital and functional T cells can be harvested from TdLNs compared to tumor-infiltrating lymphocytes (TILs), which are mostly exhausted and therefore not suitable for co-culture studies requiring the maintenance of a defined T cell to cancer cell ratio. Our allograft model was used as a tool to generate tumor-educated T cells for co-culture and we found that subcutaneous KPC PDAC tumors resemble orthotopic tumors by showing similar histology, desmoplastic TME and growth behavior^16^. Accordingly, T cells from patient-matched PBMCs will be used for co-culture with human PDOs in future studies.

Given the fact that PDOs are an essential part of a personalized medicine approach, there is still room for improvements. While more closely representing the molecular characteristics and heterogeneity of patient tumors than traditional immortalized cancer cell lines, PDO models are inherently limited in their ability to reflect the tumor microenvironment in vitro as they are comprised mostly of epithelial cells^14^. The lack of stromal cells, cancer-associated fibroblast (CAFs), immune cells and others in PDO models poses a major problem as these tumor microenvironmental components contribute to the various hallmarks of cancer and response to therapy^13,33^. Moreover, current generation PDO dome cultures are embedded in Matrigel, which show (i) inconsistency in manufacturing with batch-to-batch variability, (ii) complexity in composition, (iii) lack of reproducibility, and (iv) lack of consideration of patient-specific matrix composition^34,35^. In particular, the inability to fully recapitulate the biochemical and biophysical microenvironment considerably impede the fidelity of current preclinical PDO models in reliably predicting chemo- and immunotherapy treatment response in patients^11,12^. Based on the scaffold-free agarose microwell-array setup in our model system, target cells are first cultured in a matrix-free environment before being embedded in a customizable collagen matrix. This resulted in rapid formation of multiple uniformly-sized organoids. Previous studies showed the robustness of microwell array settings under Matrigel-free or -reduced conditions in acquiring essential organoid features, such as comparable morphology, cell differentiation capability and expression profiles matching with classical Matrigel-embedded organoid dome cultures^34,36,37^. Here we confirmed that PDOs cultures in our microwell system expressed PDAC organoid markers, such as Ck19, Ki67 and E-Cadherin (Fig. 3F, Supplementary Fig. S7), thus confirming the maintenance of the original organoid nature in our model system. Notably, we observed comparable chemotherapy response rates of the PDO lines in the InterOMaX to that of classical dome cultures (manuscript in preparation), which further validates the suitability of this platform for entering clinical translation. One of the major advantages of the InterOMax compared to co-culture setups based on domes cultures or ultra-low attachment plates combined with low percentage Matrigel/culture medium mixes^16^, is the accurate and reproducible analysis of T cell infiltration into each individual organoid. Since PDOs in the InterOMaX platform are located in the same optical plane and in well-defined distances from each other, the number and depth of infiltrated T cells can be reliably quantified in a high-throughput and automated fashion. In contrast, PDOs in dome culture differ in size and are randomly located within the Matrigel dome, thus significantly impede accuracy of T cell infiltration analysis (Fig. 4D,E). Since the Matrigel dome is attached to the bottom of the dish, PDOs face distinct stiffness gradients from top to bottom^38^. As distinct traction forces can significantly impact intracellular signaling, cell behavior and drug response, it is essential to maintain homogeneous traction forces throughout the entire PDO microenvironment to draw any conclusions from a given matrix composition.

To mimic key components of the PDAC microenvironment with the InterOMaX platform, we challenged the system by creating a multicellular co-culture setting including PDOs, T cells and hPSC. PSCs are major contributors to TME remodeling and desmoplasia^39^ and alter collagen density and alignment. Moreover, PSCs are implicated in supporting tumor growth and progression by crosstalk with PDOs. Similar to previous studies, our data suggest hPSC-mediated inhibition of effector T cell functionality in co-culture with PDOs, thus showing the feasibility of modeling complex co-culture settings and reliability in differentiating between distinct phenotypes dependent on cell–cell and cell–matrix interactions. Future studies will investigate the underlying mechanisms of these cellular interactions and differentiate an hPSC-mediated effect on matrix stiffness from a direct hPSC-PDO interaction that inhibits T cell effector functionality. Moreover, by incorporating hPSCs we intended to evaluate the technical feasibility of performing multicellular co-culture studies and enabling of cellular interactions between the different cell populations within our system. For future studies, we will incorporate CAFs in our co-culture system, since they present the most abundant cell type in the PDAC TME^40^.

**Figure 6:**
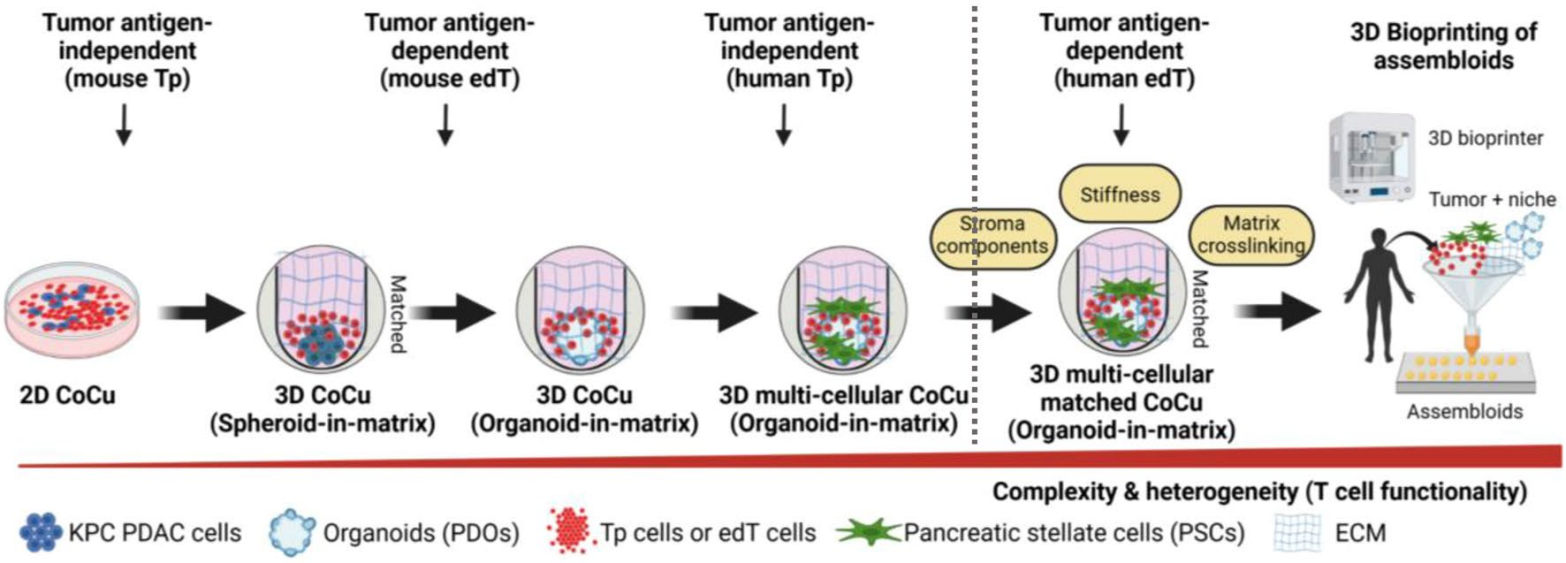
Graphical abstract. Evolution of the InterOMaX (INTERaction with Organoid-in-MatriX) platform. Schematic overview of the step-wise advancement of the 3D co-culture model system. Depicted is the increase in dimensional complexity and cellular heterogeneity from 2D adherent (murine) spheroids to 3D multicellular (human) organoid cultures. Future plans beyond the dotted line are the recreation of tumor-niche interplay using patient-matched assembloids and ECM components created with automated 3D bioprinted technologies. *PDO* patient-derived organoids, *CoCu* co-culture, *ECM* extracellular matrix, *Tp* Tumor naïve T cells from mouse spleen are activated via plate-coated Cd3e/Cd28 antibodies, *EdT* Tumor-educated T cells from tumor-draining lymph nodes (TdLNs) of subcutaneous (s.c.) allograft tumor-bearing mice.

In summary, the InterOMaX platform provides the foundation for more complex model systems and builds a bridge between investigation of individual cell–cell and cell–matrix crosstalk and 3D bioprinting technologies to create patient-derived assembloids as biological avatars (Fig. 6). The emerging understanding of the tumor-niche interplay will foster the entrance into clinical translation for drug response testing and therapy guidance.

### Limitations

Future studies will utilize a larger variety of customized matrix compositions with different stiffness grades and biochemical compositions that allow flexible fine-tuning of the matrix. Methods to quantify mechanical stiffness of the matrix e.g. by utilizing oscillatory shear rheology^41^ will considerably improve our model system for effective assessment of the biophysically and physiologically relevant microenvironment. Since the readouts only provide end point analysis so far, the platform will benefit from integration of light sheet fluorescent microscopy, particularly to monitor the dynamic behavior of T cells in real time and distinguish between distinct T cell subpopulations. Our preliminary data showed that immunofluorescent staining of infiltrated T cells in the collagen matrix using antibodies did not provide sufficient detection sensitivity due to the overall scarcity of infiltrated T cells into each spheroid or organoid. Single cell RNAseq or spatial transcriptomic analysis, e.g. by GeoMx Digital Profiler (NanoString), might be more feasible methods to identify functionally distinct T cell subpopulations in the matrix. A limitation of our Casp3/7 assay is the lack of distinction between apoptosis of infiltrated T cells and PDAC cells. This issue could be addressed by utilizing flow cytometry–based Annexin V/PI assay, in which the CD3-positive T cells can be gated out. Furthermore, this assay helps to discriminate different stages of apoptosis of target cells. Since we focused on PDAC cell-intrinsic mechanisms modulating a T cell response in our present studies, the source of T cells was kept constant for co-culture with distinct PDAC cell populations. Future studies will explore the impact of different T cell states on PDAC cytotoxicity in our co-culture platform, and will benefit from employing patient-derived cells to support interrogation of complex in vivo interactions.

## Conclusions

We report on the development of the InterOMaX platform as a versatile tool integrating multiples readouts in a comprehensive setting, and presenting a flexible framework for broad implementation of 3D culture interaction-based studies on immune response in solid tumors. This co-culture platform enables rapid, controlled and reproducible mechanistic studies, including identification and validation of candidates of T cell resistance. This allows deconvoluting complex in vivo tumor-niche interactions and sets the stage for entering clinical translation for effective decision-making and therapy-guidance.

### Supplementary Information

Supplementary Information.

## Data Availability

The datasets generated and analyzed during the current study are available in the NCBI Sequence Read Archive (SRA), Accession number PRJNA1020447 (https://www.ncbi.nlm.nih.gov/sra/PRJNA1020447).
