# Supplementary material for: A pancreatic cancer organoid-in-matrix platform shows distinct sensitivities to T cell killing
Source: Sci Rep. 2024 Apr 23;14:9377. doi: 10.1038/s41598-024-60107-5 (PMC11039640; doi:10.1038/s41598-024-60107-5)
Supplement: Supplementary file 1 — Supplementary Information. [file 41598_2024_60107_MOESM1_ESM.pdf]

A Pancreatic Cancer Organoid-in-Matrix Platform Shows Distinct Sensitivities to T cell Killing

Anton Lahusen, Jierui Cai, Reinhold Schirmbeck, Anton Wellstein, Alexander Kleger, Thomas Seufferlein, Tim Eiseler, Yuan-Na Lin

Supplementary Data

Figure S1

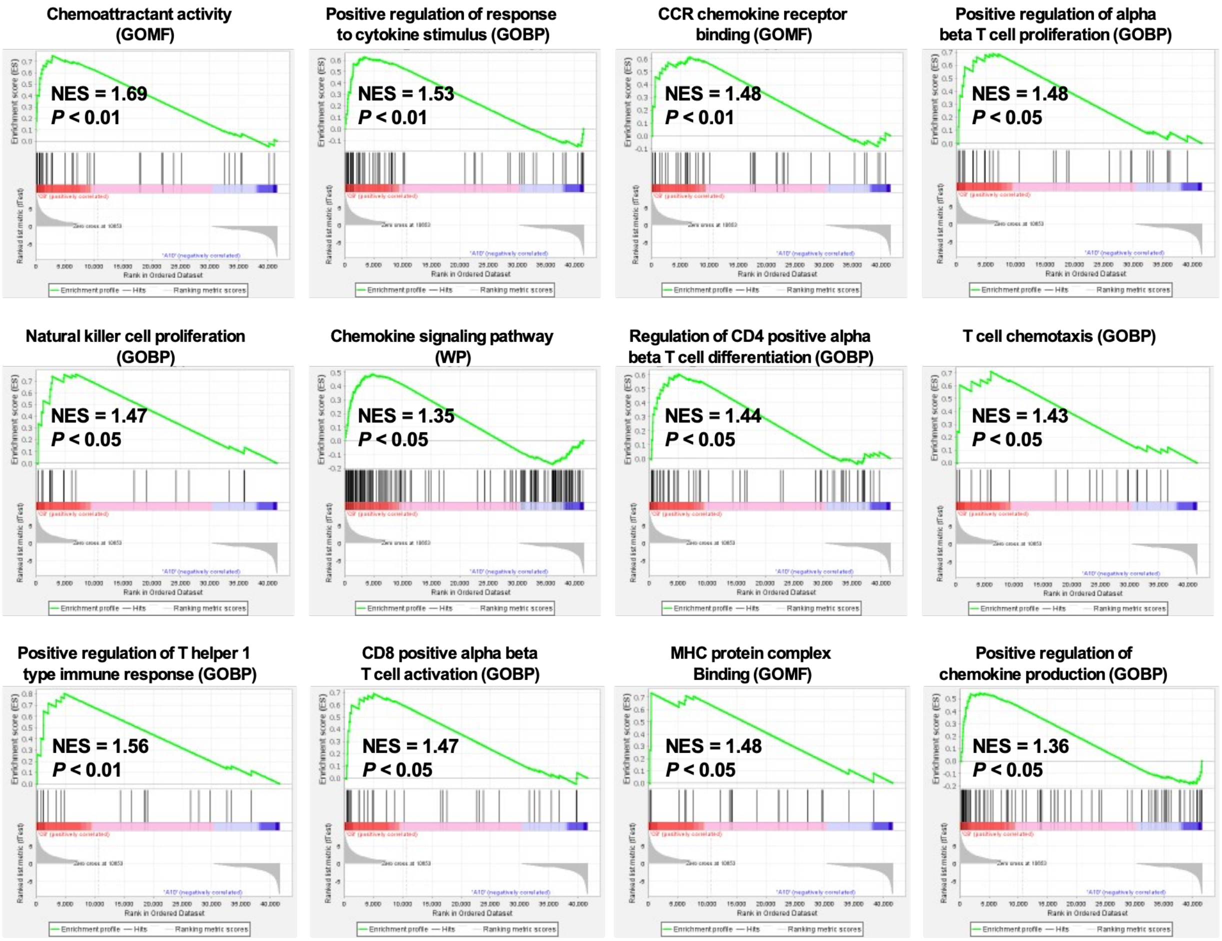

Figure S1. Enrichment of immune-relevant pathways in T cell-sensitive KPC PDAC cells. Immunologically relevant pathways (as indicated) are enriched in sensitive compared to resistant KPC PDAC cells. GSEA software was used to generate plots and enrichment scores. The analyzed gene sets originate from either GO biological pathways (GOBP), WikiPathways (WP), or GO molecular functions (GOMF) databases.

Figure S2

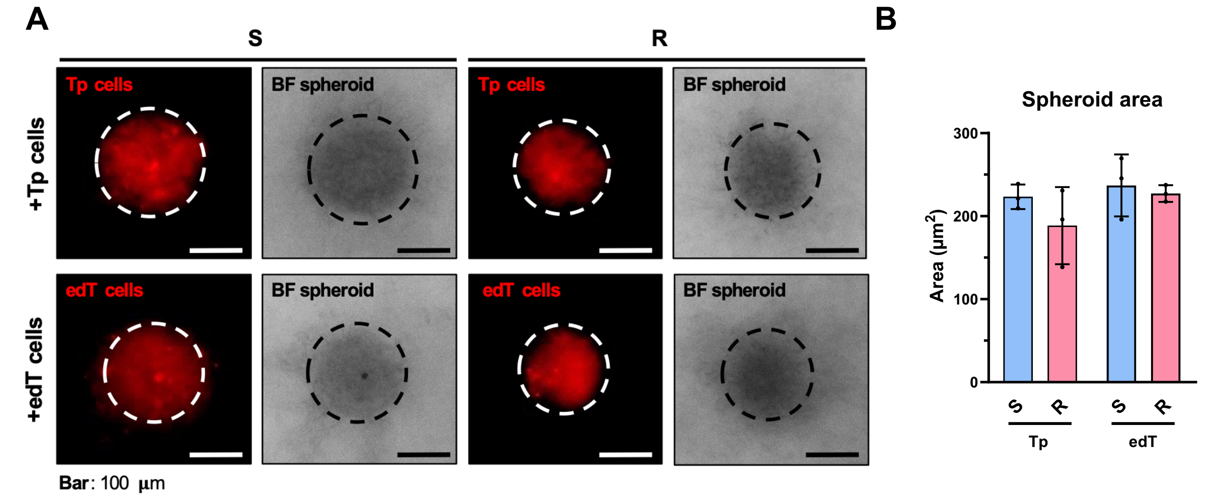

Figure S2. Area size between R- and S-KPC PDAC spheroids. (A) Representative fluorescence and brightfield (BF) images showing Cd3e/Cd28 plate-activated T (p) cells or *in vivo* tumor-educated (ed) T cells infiltrating into collagen type I matrix with resistant (R) or sensitive (S) KPC spheroids after co-culture (CoCu) for 72h. Detection of CellTracker™ Deep Red-stained T cells by fluorescence microscopy. The spheroid core area is indicated by a white (fluorescence image) or black (BF image) dashed line, respectively. The spheroid area overlaps with the background fluorescence signal from the Cy5 channel. (B) Quantification of the spheroid area displayed in (A). N = 3.

Figure S3

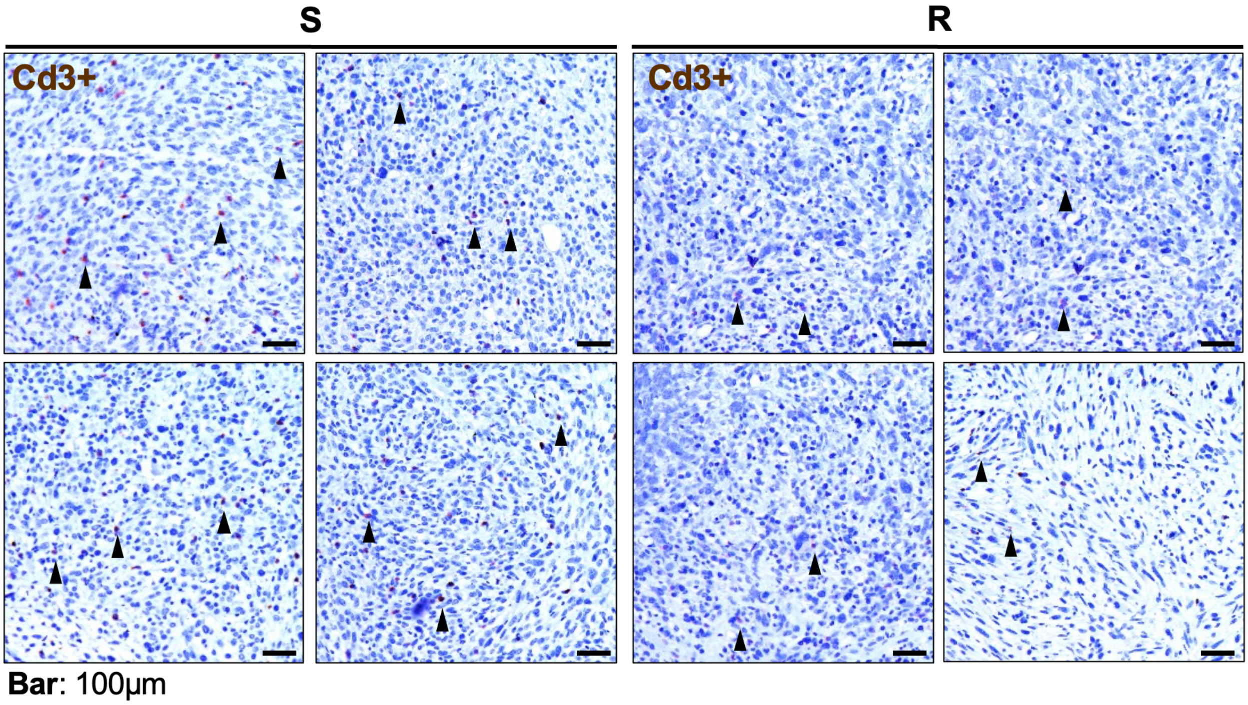

Figure S3. Increased infiltration of T cells into sensitive (S) compared to resistant (R) subcutaneous tumor tissues. Infiltration of Cd3 positive (+) T cells into subcutaneous allograft tumors from S- and R- KPC PDAC clonal cell lines. T cells were detected by DAB staining immunohistochemistry and pictures were taken at 10X magnification. Infiltrating Cd3+ T cells are indicated by black arrows. N = 4.

Figure S4

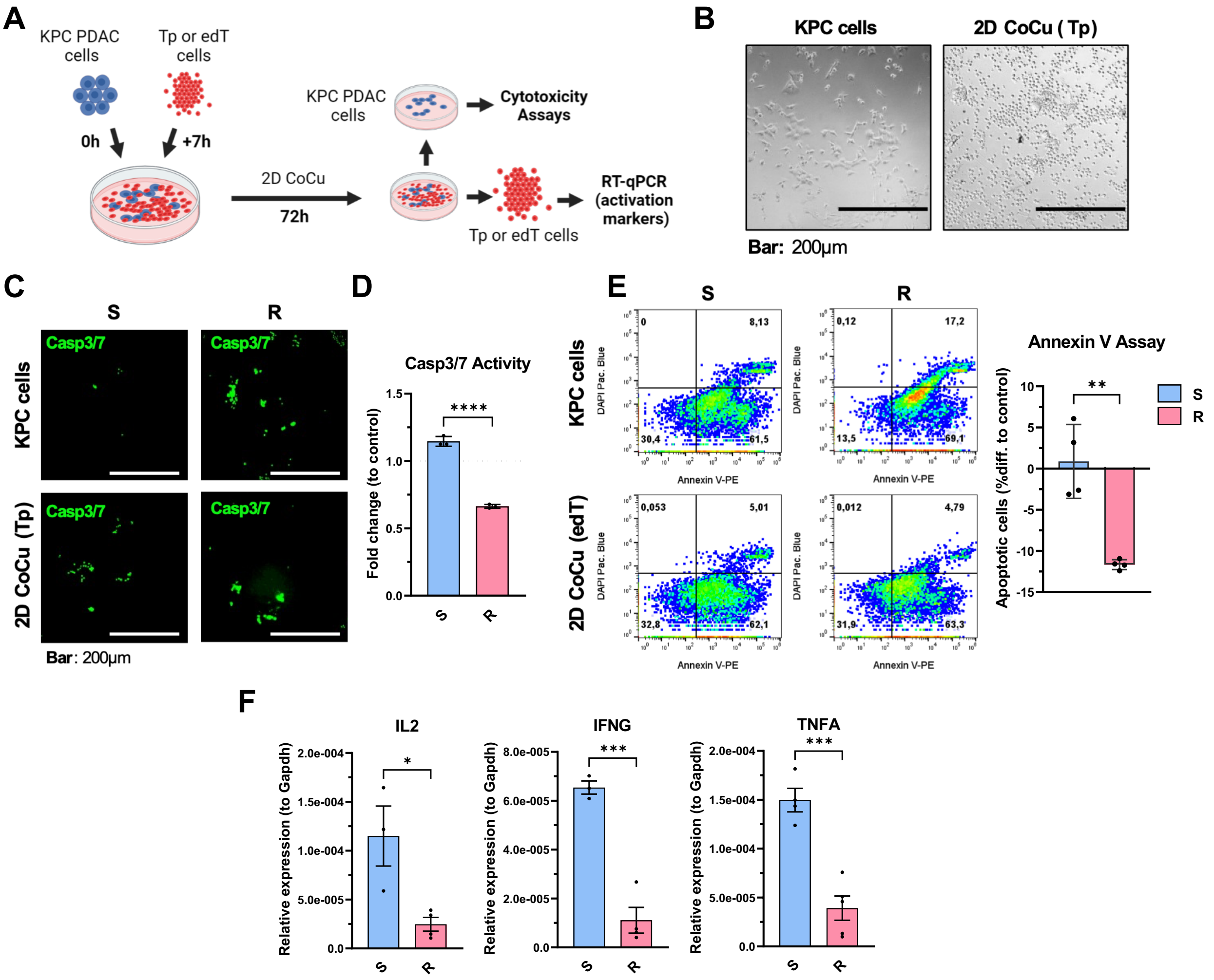

Figure S4. 2D co-culture screening of KPC PDAC immune response phenotypes. (A) Schematic overview of the 2D co-culture (CoCu) workflow. Tp or edT cells were added to adherent cancer cells. Tp: Tumor naïve T cells from mouse spleen activated via plate-coated Cd3e/Cd28 antibodies. EdT: Tumor-educated T cells from tumor-draining lymph nodes (TdLNs) of subcutaneous (s.c.) allograft tumor-bearing mice. Wt: wild-type mice. (B) Brightfield images (10X magnification) of KPC PDAC cells grown for 72h without (control) or with (2D CoCu) Tp cells. (C) Representative fluorescence images (10X magnification) showing Casp3/7-positive resistant (R) or sensitive (S) KPC cells grown for 72h in adherent culture without (KPC cells only control) or with (2D CoCu) Tp cells. Casp3/7 activity was determined using the CellEvent™ Caspase3/7 Detection Reagent. (D) Number of fluorescence-positive cells were counted per area by using the Fiji Macro. Means were calculated for six areas respectively. Results are shown as fold change to KPC only control. N = 3. (E) Annexin V Assay for the identification of late apoptosis in sensitive (S) or resistant (R) KPC cells grown for 72h without (KPC only control) or with (2D CoCu) edT cells. DAPI- and Annexin V PE-stained single cells were analyzed by flow cytometry. Late apoptotic cells show DAPI/Annexin V-PE double positive staining. Unstained samples were used as gating control. For quantification, the difference in percentage of apoptotic KPC cells in 2D edT cell co-culture, and apoptotic KPC cells only control was calculated. N = 4. (F) Relative expression ($2^{-\Delta Ct}$) via RT-qPCR of the given T cell activation markers in Tp cells after 72h co-culture with resistant (R) or sensitive (S) KPC PDAC cells. Results were normalized to *GAPDH*. N ≥ 3 independent biological replicates. * $p \leq 0.05$, ** $p \leq 0.01$, *** $p \leq 0.001$, **** $p \leq 0.0001$.

Figure S5

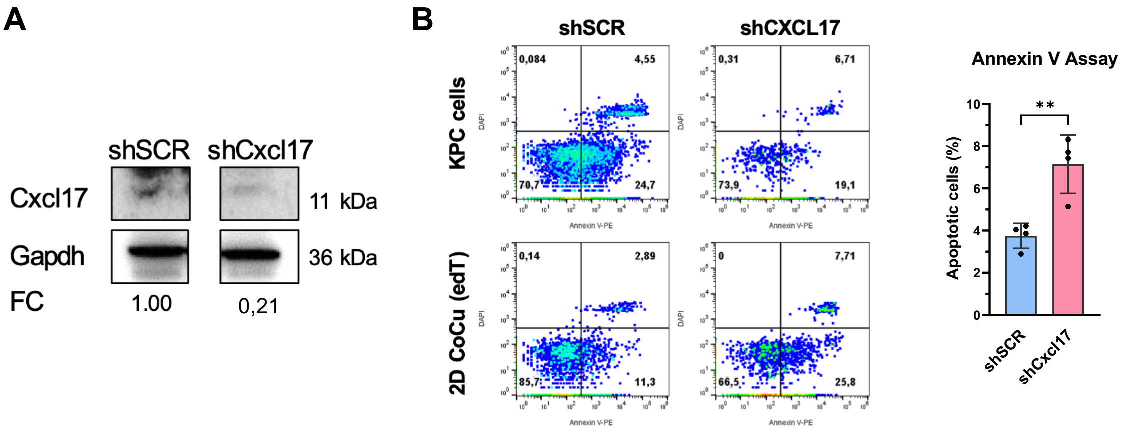

Figure S5. Knock down of CXCL17 in resistant KPC PDAC clonal cells increases T cell-mediated cytotoxicity. (A) The CXCL17 knock down was confirmed by Western blot analysis (11 kDa) or *GAPDH* (36 kDa) and by the quantification of band densities using the BandPeakQuantification ImageJ macro. Band densities were normalized to *GAPDH* and are shown as fold downregulated relative to scrambled control (shSCR). (B) Late apoptosis in control (shSCR) or CXCL17 knock down cells (shCxcl17) co-cultured for 72h with edT cells (2D CoCu) by Annexin V assay. DAPI- and Annexin V PE-stained single cells were analyzed by flow cytometry. Late apoptotic cells showing DAPI/Annexin V-PE double positive staining. Unstained samples were used as gating control. N = 4 independent biological replicates. ** $p \leq 0.01$.

Figure S6

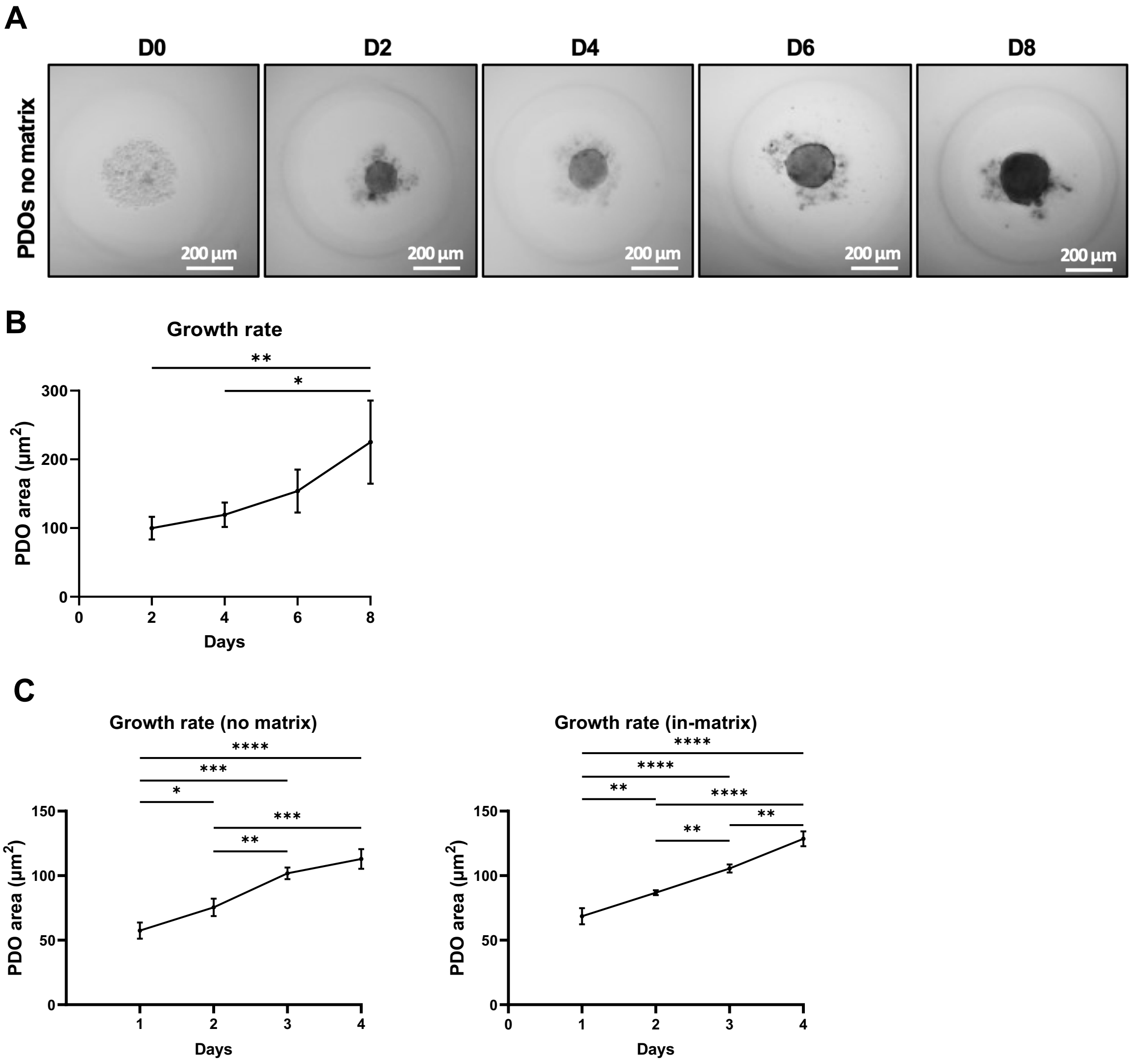

Figure S6. Stable growth of PDOs in human feeder medium in 3D hydrogel chips over time. (A) Representative brightfield images (10X magnification) of PDOs grown without collagen matrix in hydrogel chips for 8 days (D0-D8). (B) The growth rate was quantified by measuring PDO area per time point. N=8 independent replicates. (C) PDO growth rates (no matrix and in-matrix, days D1-D4, respectively) were quantified by measuring PDO area per time point. N = 3 independent biological replicates. * $p \leq 0.05$, ** $p \leq 0.01$, *** $p \leq 0.001$, **** $p \leq 0.0001$.

Figure S7

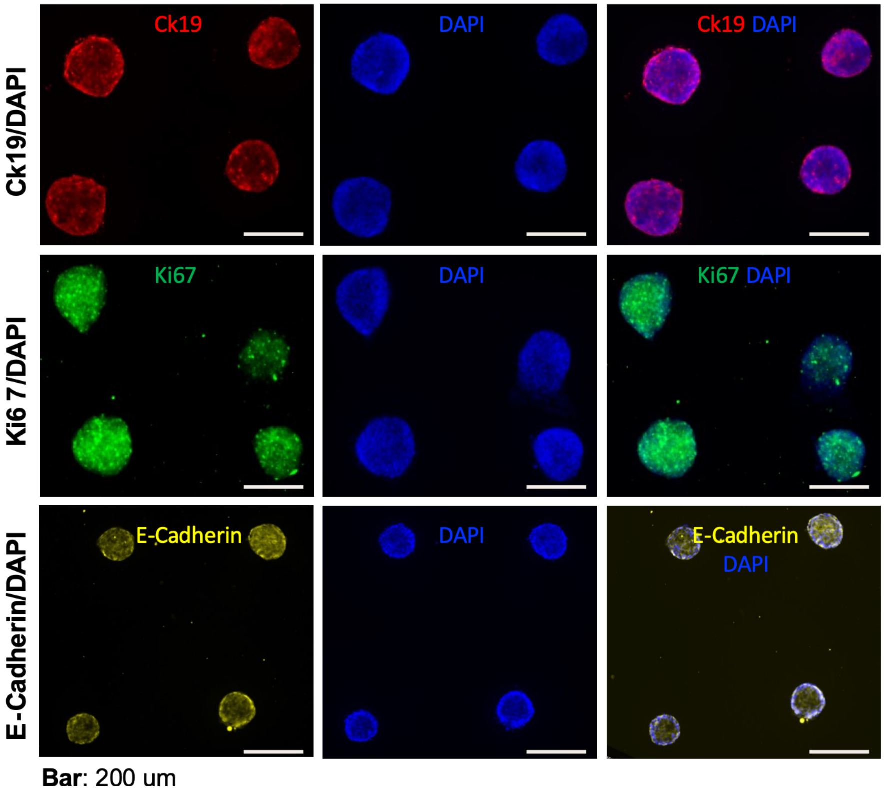

Figure S7. Immunofluorescence staining of human PDOs-in-matrix for PDAC PDO-specific markers. Representative fluorescence microscopy images for immunofluorescence staining of the indicated PDO markers Ck19, Ki67, and E-Cadherin (4X magnification). Markers are shown as single stain and merge with DAPI respectively. PDOs were grown for 72h in 3mg/ml collagen type I matrix on hydrogel chips prior to the staining procedure. N=3 independent biological replicates.

Figure S8

```
// Automated fluorescent cell counter, use 10X pictures, remove background prior to analysis

macro "Cell_count--Threshold" {

//show prompt for selection of source directory
dir = getDirectory("Choose source directory");
list = getFileList(dir);	//get the file list
Array.sort(list); // bypass of array "order bug" on some processors
resultsFileName = "Migration_Cell_Count.txt"

File.makeDirectory(dir+"Processed_images"); //make directory to store processed images

setBatchMode(true);	//hide all the details from user
//process every file...
	for (i=0; i<list.length; i++) {

		//...that has .tif extension
		if (endsWith(list[i], ".tif")) {

		open(dir+list[i]);
		fileNoExt = split(list[i], ".");

			run("Subtract Background...", "rolling=50");

			run("Color Threshold...");
			// Color Thresholder 1.50i
			// Autogenerated macro, single images only!
			min=newArray(3);
			max=newArray(3);
			filter=newArray(3);
```

```
a=getTitle();
run("HSB Stack");
run("Convert Stack to Images");
selectWindow("Hue");
rename("0");
selectWindow("Saturation");
rename("1");
selectWindow("Brightness");
rename("2");
min[0]=0;
max[0]=255;
filter[0]="pass";
min[1]=0;
max[1]=255;
filter[1]="pass";
min[2]=0;
max[2]=100;
filter[2]="pass";
for (z=0;z<3;z++){
	selectWindow(""+z);
	setThreshold(min[z], max[z]);
	run("Convert to Mask");
	if (filter[z]=="stop")  run("Invert");
}
imageCalculator("AND create", "0","1");
imageCalculator("AND create", "Result of 0","2");
	for (z=0;z<3;z++){
		selectWindow(""+z);
		close();
	}
selectWindow("Result of 0");
close();
```

```
			selectWindow("Result of Result of 0");
			rename(a);
			// Colour Thresholding


			run("Convert to Mask");//convert to mask and analyze
			run("Convert to Mask");
			run("Watershed");


			run("Analyze Particles...", "size=10-200 pixel circularity=0.00-1.00
show=Outlines clear exclude summarize");


				// save the file in a new directory under a new name and close all
windows
			saveAs("Jpeg",
dir+"Processed_images"+File.separator+"Analysis_of_"+fileNoExt[0]+"."+fileNoExt[1]);
			close();


			selectWindow(list[i]);
			close();


		}


	}
				//copy all relevant contents from the Summary window
			selectWindow("Summary");
			text = getInfo("window.contents");
			lines = split(text, "\n");


				//create a text file with counting results, output only cell count
				//copy the results into clipboard
			String.resetBuffer;		//reset string buffer
			for (i=0; i<lines.length; i++) {
				if (i==0){
```

```
                File.saveString("",dir+resultsFileName);
                f = File.open(dir+resultsFileName);
            }
            labels = split(lines[i], "\t");
            print(f,labels[1]);
            String.append(labels[1]+"\n");    //append another value to the string

        }
        File.close(f);



            //open the summary file, close the Summary window
        open(dir+resultsFileName);
        selectWindow("Summary");
        run("Close");
        String.copy(String.buffer);           //copy all values into the clipboard

setBatchMode(false);

}
```

Figure S9

```
Dialog.create("Band/Peak Quantification Tool");
Dialog.addNumber("background width (pixels)", 3);
Dialog.addChoice("Estimate background from (rectangle selection only): ", newArray("all",
"top/bottom", "sides"));
Dialog.addChoice("Background estimation by: ", newArray("median", "mean"));
Dialog.addCheckbox("Reset scale", true);
Dialog.show();

// Check selection shape
if (roiManager("count") == 0) {
	type=selectionType();
} else {
	roiManager("Select", 0);
	type=selectionType();
}

// Get dialog options
expand=Dialog.getNumber();
backpos=Dialog.getChoice();
backtype=Dialog.getChoice();
resetscale=Dialog.getCheckbox();

// Reset scale, if scale is set for the image (without this block, this macro gives error when "scaled"
image is used)
if (resetscale) {
	run("Set Scale...", "distance=0 known=0 pixel=1 unit=pixel");
}

// Warn if top/bottom or sides option was selected for non-rectangular selections
if ((backpos != "all") && (type != 0)) {
```

```
		print("'Top/bottom' or 'sides' can be only applicable to rectangle selections. 'All' is used instead.");
}


// Main
if ((backpos == "all") | (type != 0)){
if (roiManager("count") == 0) {
		Roi.getBounds(x,y,w,h);
		getStatistics(area, mean);
		run("Make Band...", "band="+expand);
		if (backtype == "median") {
			mean_back=getValue("Median");
		} else {
			mean_back=getValue("Mean");
		}
		i=nResults();
		setResult("signal", i, area*(mean-mean_back));
		setResult("total", i, area*mean);
		setResult("area", i, area);
		setResult("mean", i, mean);
		if (backtype == "mean"){
			setResult("mean_background", i, mean_back);
		} else {
			setResult("median_background", i, mean_back);
		}
		setResult("ROI_x", i, x);
		setResult("ROI_y", i, y);
		setResult("ROI_w", i, w);
		setResult("ROI_h", i, h);
		updateResults();
		makeRectangle(x, y, w, h);
} else {
```

```
		for (k=0; k < roiManager("count"); k=k+1) {
			roiManager("Select", k);
			Roi.getBounds(x,y,w,h);
			getStatistics(area, mean);
			run("Make Band...", "band=3");
			if (backtype == "median") {
				mean_back=getValue("Median");
			} else {
				mean_back=getValue("Mean");
			}
			i=nResults();
			setResult("signal", i, area*(mean-mean_back));
			setResult("total", i, area*mean);
			setResult("area", i, area);
			setResult("mean", i, mean);
			if (backtype == "mean"){
				setResult("mean_background", i, mean_back);
			} else {
				setResult("median_background", i, mean_back);
			}
			setResult("ROI_x", i, x);
			setResult("ROI_y", i, y);
			setResult("ROI_w", i, w);
			setResult("ROI_h", i, h);
			updateResults();
			roiManager("Select", k);
		}
	}
	} else {
	if (backpos == "top/bottom" ){
	if (roiManager("count") == 0) {
		Roi.getBounds(x,y,w,h);//Note that x and y denotes top-lefts point
```

```
		getStatistics(area, mean);
		makePolygon(x, y-expand, x+w, y-expand,
						x+w, y, x, y,
						x, y+h, x+w, y+h,
						x+w, y+h+expand, x, y+h+expand,
						x, y-expand);
		if (backtype == "median") {
			mean_back=getValue("Median");
		} else {
			mean_back=getValue("Mean");
		}
		i=nResults();
		setResult("signal", i, area*(mean-mean_back));
		setResult("total", i, area*mean);
		setResult("area", i, area);
		setResult("mean", i, mean);
		if (backtype == "mean"){
			setResult("mean_background", i, mean_back);
		} else {
			setResult("median_background", i, mean_back);
		}
		setResult("ROI_x", i, x);
		setResult("ROI_y", i, y);
		setResult("ROI_w", i, w);
		setResult("ROI_h", i, h);
		updateResults();
		makeRectangle(x, y, w, h);
	} else {
		for (k=0; k < roiManager("count"); k=k+1) {
			roiManager("Select", k);
			Roi.getBounds(x,y,w,h);
			getStatistics(area, mean);
```

```
					makePolygon(x, y-expand, x+w, y-expand,
							x+w, y, x, y,
							x, y+h, x+w, y+h,
							x+w, y+h+expand, x, y+h+expand,
							x, y-expand);
					if (backtype == "median") {
						mean_back=getValue("Median");
					} else {
						mean_back=getValue("Mean");
					}
					i=nResults();
					setResult("signal", i, area*(mean-mean_back));
					setResult("total", i, area*mean);
					setResult("area", i, area);
					setResult("mean", i, mean);
					if (backtype == "mean"){
						setResult("mean_background", i, mean_back);
					} else {
						setResult("median_background", i, mean_back);
					}
					setResult("ROI_x", i, x);
					setResult("ROI_y", i, y);
					setResult("ROI_w", i, w);
					setResult("ROI_h", i, h);
					updateResults();
					roiManager("Select", k);
				}
			}
			} else {// sides
			if (roiManager("count") == 0) {
				Roi.getBounds(x,y,w,h);
				getStatistics(area, mean);
```

```
        makePolygon(x-expand, y, x-expand, y+h,
                    x, y+h, x, y,
                    x+w, y, x+w, y+h,
                    x+w+expand,y+h, x+w+expand, y,
                    x-expand, y);
        if (backtype == "median") {
            mean_back=getValue("Median");
        } else {
            mean_back=getValue("Mean");
        }
        i=nResults();
        setResult("signal", i, area*(mean-mean_back));
        setResult("total", i, area*mean);
        setResult("area", i, area);
        setResult("mean", i, mean);
        if (backtype == "mean"){
            setResult("mean_background", i, mean_back);
        } else {
            setResult("median_background", i, mean_back);
        }
        setResult("ROI_x", i, x);
        setResult("ROI_y", i, y);
        setResult("ROI_w", i, w);
        setResult("ROI_h", i, h);
        updateResults();
        makeRectangle(x, y, w, h);
    } else {
        for (k=0; k < roiManager("count"); k=k+1) {
            roiManager("Select", k);
            Roi.getBounds(x,y,w,h);
            getStatistics(area, mean);
            makePolygon(x-expand, y, x-expand, y+h,
```

```
				x, y+h, x, y,
				x+w, y, x+w, y+h,
				x+w+expand,y+h, x+w+expand, y,
				x-expand, y);
		if (backtype == "median") {
			mean_back=getValue("Median");
		} else {
			mean_back=getValue("Mean");
		}
		i=nResults();
		setResult("signal", i, area*(mean-mean_back));
		setResult("total", i, area*mean);
		setResult("area", i, area);
		setResult("mean", i, mean);
		if (backtype == "mean"){
			setResult("mean_background", i, mean_back);
		} else {
			setResult("median_background", i, mean_back);
		}
		setResult("ROI_x", i, x);
		setResult("ROI_y", i, y);
		setResult("ROI_w", i, w);
		setResult("ROI_h", i, h);
		updateResults();
		roiManager("Select", k);
	}
}
}
}
```

Figure S10

```
// To run this script, you need to first save screen grab of your tiff.
// This script is set up for 50-66% zoom in OliVia.
// Then click Run when you're ready and wait for prompts.

// splits images to other colors
run("Set Measurements...", "area limit redirect=None decimal=3");
run("Colour Deconvolution", "vectors=[H DAB]");
waitForUser("Image selection", "Please select the brown image as have it as the front image. Then Click 'OK'.")

// change brown image to b/w
run("8-bit");
run("Invert");

setTool("polygon");
waitForUser("Exluding areas you don't want", "Please draw around the area you want to exlude. Then Click 'OK'.")
roiManager("Add")
run("Clear", "slice");
roiManager("Deselect")
run("Select All");

run("Subtract...", "value=50");
setAutoThreshold("Default dark");

// Thresholding. Try and cover as much as you can, to the point of overestimating.
run("Threshold...");
setOption("BlackBackground", false);
waitForUser("Thresholding", "Please drag the red threshold until you have covered the objects you want to include as precise as you can. Then Click 'OK'.")
```

```
// Convert to binary and fill holes + split cells.
run("Convert to Mask");
run("Close-");
run("Fill Holes");
run("Watershed");

// You may need to adjust the "size=..." value if you use a differently sized image. It is currently set up for 66% zoom.
// If you want to use a lower magnification image, you can decrease the min size accordingly
run("Analyze Particles...", "size=100-3000 show=[Overlay Masks] display exclude clear summarize");

// Check your original image with the overlay.
// If you're not happy, redo the thresholding, or adjust the size in the script.
// Count number of cells in field of analysis, exlcuding the region excluded earlier.
waitForUser("Select blue channel", "Please and select the blue channel NOW. Click 'OK' when ready.")
run("8-bit");
run("Invert");
run("Subtract...", "value=20");
roiManager("Select", 0);
setBackgroundColor(0, 0, 0);
run("Clear", "slice");
roiManager("Deselect");
run("Select All");

setAutoThreshold("Default dark");

run("Threshold...");
waitForUser("Threshold the nuclei", "Drag the red threshold until you have covered the nuclei as precise as you can. Click 'OK' when ready.")
run("Convert to Mask");
run("Close-");
run("Fill Holes");
```

```
run("Watershed");


run("Set Measurements...", "limit redirect=None decimal=3");

run("Analyze Particles...", "size=100-3000 show=[Overlay Masks] display exclude summarize");


// Copy the resulting table to excel.

waitForUser("Last", "Cross check with original image and ammend the cell count accordingly. Copy results table to spreadsheet. Click 'OK' to close all. Seeya and thank you!")

run("Close All");

roiManager("Delete");
```

Table S1

Human-wash-medium

Reagent	Stock conc.	Final conc.	Dilution factor	Volume [500 mL]	Note
Advanced DMEM/F-12	´-	´-	´-	489 mL	
HEPES	1 M	10 mM	100	5 mL	
GlutaMAX	100x	1x	100	5 mL	
Primocin	50 mg/mL	100 µg/mL	500	1 mL	PenStrep is used (Boj et al.)

Human-complete-feeding-medium (hCPLT) (for 100 mL)

Reagent	Stock-conc.	Final conc.	Dilution factor	Volume [100 mL]	Volume [200 mL]	Volume [300 mL]	Note
Human-Wash-medium	-	-		36.4 mL	72.8 mL	109.2 mL	
WNT3A-CM* (50%)	2x	1x	2	50 mL	100 mL	150 mL	
R-spondin1-CM* (10%)	10x	1x	10	10 mL	20 mL	30 mL	
B27 supplement	50x	1x	50	2 mL	4 mL	6 mL	
Nicotinamide	1 M	10 mM	100	1 mL	2 mL	3 mL	
N-acetylcysteine	500 mM	1,25 mM	400	250 µL	500 µL	750 µL	Boj et al., 1 mM
Primocin	50 mg/mL	100 µg/mL	500	200 µL	400 µL	600 µL	Boj et al., 1 mg/mL
mNoggin	100 µg/mL	100 ng/mL	1000	100 µL	200 µL	300 µL	
hEGF	500 µg/mL	50 ng/mL	10.000	10 µL	20 µL	30 µL	
hFGF10	1 mg/mL	100 ng/mL	10.000	10 µL	20 µL	30 µL	
hGastrin I	100 µM	10 nM	10.000	10 µL	20 µL	30 µL	
A 83-01	25 mM	500 nM	50.000	2 µL	4 µL	6 µL	
Y27632 Rock-Inhibitor**	10 mM	10 µM	1000				

*CM=Conditioned Medium **everytime fresh

Reagent	Manufacturer	Reference
Advanced DMEM/F-12	Life Technologies	12634028
HEPES	Lonza	17-737E
GlutaMAX	Gibco	35050-038
Primocin	InvivoGen	ant-pm-2

Reagent	Manufacturer	Reference
Human-Wash-medium	See above	-
WNT3A-CM*	Conditioned medium	-
R-spondin1-CM*	Conditioned medium	-
B27 supplement	Thermo Fisher Scientific	12587-010
Nicotinamide	Sigma-Aldrich	N0636
N-acetylcysteine	Sigma-Aldrich	A9165
Primocin	InvivoGen	ant-pm-2
mNoggin	Peprotech	250-38
hEGF	Peprotech	AF-100-15
hFGF10	Miltenyi Biotec	130-127-857
hGastrin I	Tocris	3006
A 83-01	Tocris	2939/10
Y27632 Rock-Inhibitor	Stem Cell	72305

Table S1: PDO media composition. Modifications from the original protocol from Boj et al.[24] are indicated.

Table S2

Target Species	Target Gene	GeneGlobe ID (#249900 Qiagen)
Mouse	Il2	QT02521309
Mouse	Ifng	QT01038821
Mouse	Gzmb	QT00114590
Mouse	Tnfa	QT00104006
Mouse	Gapdh	QT01658692
Human	IL2	QT00015435
Human	IFNG	QT00000525
Human	GZMB	QT01001875
Human	EOMES	QT00026495
Human	FOXP3	QT00048286
Human	GAPDH	QT02504278

Table S2: Primers from Qiagen (#249900) used undiluted for qRT-PCR.
